# Binder-Free Immobilization of Photocatalyst on Membrane Surface for Efficient Photocatalytic H_2_O_2_ Production and Water Decontamination

**DOI:** 10.1007/s40820-025-01822-0

**Published:** 2025-06-18

**Authors:** Zhen-Yu Hu, Tian Liu, Yu-Ru Yang, Alicia Kyoungjin An, Kim Meow Liew, Wen-Wei Li

**Affiliations:** 1 State Key Laboratory of Advanced Environmental Technology, Department of Environmental Science and Engineering, University of Science and Technology of China, Hefei, 230026 People’s Republic of China; 2 SEEM Innovation Center, Suzhou Institute for Advanced Research, University of Science & Technology of China, Suzhou, 215123 People’s Republic of China; 3 School of Resources and Environmental Engineering, Hefei University of Technology, Hefei, 230009 People’s Republic of China; 4 School of Energy and Environmental, City University of Hong Kong, Hong Kong SAR, 999077 People’s Republic of China; 5 Department of Chemical and Biological Engineering, Hong Kong University of Science and Technology, Hong Kong SAR, People’s Republic of China; 6 Department of Architecture and Civil Engineering, City University of Hong Kong, Hong Kong SAR, 999077 People’s Republic of China

**Keywords:** Photocatalytic membrane, Immobilization, Micropollutants, Water treatment, H_2_O_2_ photosynthesis

## Abstract

In photocatalytic water treatment processes, the particulate photocatalysts are typically immobilized on membrane, through either chemical/physical loading onto the surface or directly embedding in the membrane matrix. However, these immobilization strategies inevitably compromise the interfacial mass diffusion and cause activity decline relative to the suspended catalyst. Here, we propose a binder-free surface immobilization strategy for fabrication of high-activity photocatalytic membrane. Through a simple dimethylformamide (DMF) treatment, the nanofibers of polyvinylidene fluoride membrane were softened and stretched, creating enlarged micropores to efficiently capture the photocatalyst. Subsequently, the nanofibers underwent shrinking during DMF evaporation, thus firmly strapping the photocatalyst microparticles on the membrane surface. This surface self-bounded photocatalytic membrane, with firmly bounded yet highly exposed photocatalyst, exhibited 4.2-fold higher efficiency in hydrogen peroxide (H_2_O_2_) photosynthesis than the matrix-embedded control, due to improved O_2_ accessibility and H_2_O_2_ diffusion. It even outperformed the suspension photocatalytic system attributed to alleviated H_2_O_2_ decomposition at the hydrophobic surface. When adopted for UV-based water treatment, the photocatalytic system exhibited tenfold faster micropollutants photodegradation than the catalyst-free control and demonstrated superior robustness for treating contaminated tap water, lake water and secondary wastewater effluent. This immobilization strategy can also be extended to the fabrication of other photocatalytic membranes with diverse catalyst types and membrane substrate. Overall, our work opens a facile avenue for fabrication of high-performance photocatalytic membranes, which may benefit advanced oxidation water purification application and beyond.

**Supplementary Information:**

The online version contains supplementary material available at 10.1007/s40820-025-01822-0.

## Highlights

- Polyvinylidene fluoride nanofibers were treated by dimethylformamide to create micropores on membrane surface.
- Photocatalysts were firmly bounded by nanofibers during stretching and shrinking.
- Particulate catalysts were fixed yet high exposed on the membrane surface.
- Surface self-bounded photocatalytic membrane exhibited tenfold higher decontamination activity than embedded catalyst.

**Supplementary Information:**

The online version contains supplementary material available at 10.1007/s40820-025-01822-0.

## Graphical abstract

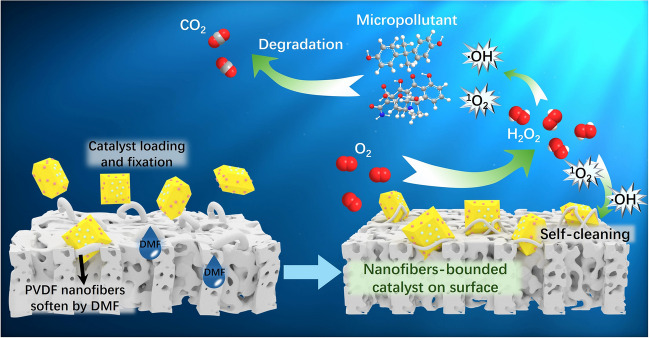

## Introduction

Water pollution by micropollutants, including various endocrine disrupting substances and antibiotics, has raised widespread ecological and health concerns [1–3]. However, low-carbon and economically affordable technologies for efficiently eliminating these micropollutants are still lacking [4]. In this respect, the UV/H_2_O_2_ advanced oxidation processes hold a great potential, due to less formation of toxic byproduct than the conventional UV/Cl_2_ processes for water disinfection and decontamination [5, 6]. In the UV/H_2_O_2_ process, the H_2_O_2_ oxidant undergoes photolysis to generate hydroxyl radicals (·OH) for pollutant degradation [7, 8]. Therefore, a continuous supply of H_2_O_2_, either through exogenous addition or through in situ generation, is needed to sustain the reaction [8–10].

Of particular interest is the in situ H_2_O_2_ generation by electrochemical or photocatalytic processes, which can avoid the complicated processes of H_2_O_2_ transportation and storage [11–13]. The technologies for H_2_O_2_ electrosynthesis have been well established to date, but their application niches have been mainly limited to saline wastewater and seawater which feature low ions migration resistance [14–17]. In comparison, H_2_O_2_ photosynthesis is adaptable to broader range of water matrix due to less restriction by solution conductivity [18–20] and allows for direct integration into UV water treatment process. Nevertheless, the limited activity of the photocatalytic systems presents a significant barrier to their practical environmental application. Especially, the immobilization of particulate photocatalyst onto a membrane substrate, which is typically essential for continuous-flow water treatment, always lead to activity decline [21–24]. In these systems, the catalyst was usually cross-linked to the membrane surface by using binders or was directly incorporated into the membrane matrix during membrane fabrication [25–29]. In either way, the active sites of catalyst were “embedded”, limiting their exposure to the bulk water and hence severely restricting the light penetration and reactant access. For example, the BiVO_4_ photocatalyst embedded in the polyvinylidene fluoride (PVDF) matrix exhibited only 50% the H_2_O_2_ photosynthesis activity of the suspended counterpart [30]. Overall, immobilizing the catalyst without compromising the performance remains a key challenge for photocatalytic membrane fabrication and water treatment application.

Here, we propose a binder-free immobilization strategy for stably fixing the catalyst particles on the PVDF surface and meanwhile improving its photocatalytic activity. Previous studies have demonstrated that some chemical agents like dimethylformamide (DMF) can soften or even dissolve PVDF membrane through weakening dipole attraction and hydrogen bond of the PVDF polymer chain [31, 32]. Therefore, we hypothesize that, by fine-regulating the DMF concentration to control the softening degree, the PVDF fibers may be stretched but without causing significant structure damage, thus creating micropores with appropriate pore sizes to hold the particulate catalyst. Then, accompanied with DMF evaporation, the PVDF fibers may undergo shrinking to strap the photocatalyst, thus firmly confining them on the membrane surface without using any chemical binders.

For validation, we adopted a mixed solution of DMF and ethanol for pre-softening the PVDF membrane prior to the photocatalyst loading via vacuum filtration. The faceted CoO_x_/Mo:BiVO_4_/Pd, a particulate inorganic photocatalyst with superior photocatalytic activity and stability for H_2_O_2_ photosynthesis [33], was adopted for the proof-of-concept study. As expected, the “stretching” and “shrinking” of the membrane fibers during DMF treatment created micropores, with dynamically changed pore sizes, to efficiently confine the photocatalyst microparticles on the membrane surface. In this way, the photocatalysts were firmly bounded yet highly-exposed on the PVDF membrane surface, forming a self-bounded photocatalytic membrane (SSPM). We show that the SSPM was not restricted by light penetration nor by mass diffusion, thus drastically improving the O_2_ accessibility and H_2_O_2_ diffusion than the conventional matrix-embedded photocatalytic membrane (MEPM). Meanwhile, it far outperformed the membrane with loosely loaded catalyst in terms of operational stability under flow water condition. The SSPM exhibited 4.2 times higher H_2_O_2_ production rate than the MEPM under simulated solar light and achieved tenfold faster UV photodegradation of tetracycline (TC) and bisphenol A (BPA) than the catalyst-free control. A superior robustness of the UV photocatalytic system for treating real water samples, including tap water, lake water and secondary wastewater effluent, was also demonstrated.

## Materials and Methods

### Chemicals

The chemicals of K_2_CO_3_, V_2_O_5,_ Bi(NO_3_)_3_·5H_2_O, Co(NO_3_)_2_, Na_2_PdCl_4_, NaIO_3_, DMF, tetracycline, bisphenol A and Ti(SO_4_)_2_ were purchased from Shanghai Aladdin Biochemical Technology Co., LTD. The PVDF membrane was purchased from Merck & Co., Inc.

### Photocatalyst Preparation

The photocatalysts were prepared following the procedures reported in a previous study [33]. Briefly, single-crystal BiVO_4_ was prepared by heating the mixture of K_2_CO_3_ (1.047 g) and V_2_O_5_ (2.272 g) in a ceramic crucible at a heating rate of 1.5 °C min^−1^ to 450 °C, followed by annealing for 5 h in a muffle furnace. The obtained K_3_V_5_O_14_ (2 g) was mixed with Bi(NO_3_)_3_•5H_2_O (0.326 g) and dispersed in 50 mL water solution under ultrasonication for 30 min. The mixture was then heated at 70 °C for 10 h under ultrasonication, separated by centrifugation, washed with deionized (DI) water, and dried at 70 °C for 8 h.

The as-prepared BiVO_4_ (0.2 g) was dispersed in 100 mL water, followed by adding 0.1 mol NaIO_3_ and 0.35 mL Co(NO_3_)_2_ solution (1.5 g L^−1^). The mixture was purged by N_2_ and irradiated for 3 h under a xenon lamp (model 300 DUV; Perfect Light, Inc., light intensity = 0.1 mW cm^−2^, λ > 420 nm), filtered, washed with DI water, and dried at 60 °C for 8 h. The as-prepared CoO_x_/BiVO_4_ (0.15 g) was dispersed in 100 mL DI water, followed by addition of 0.182 mL Na_2_PdCl_4_ solution (3.3 g L^−1^). The mixture was purged by N_2_ and irradiated at λ > 420 nm for 3 h. As-prepared CoO_x_/BiVO_4_/Pd was filtered, washed with deionized water, and dried at 60 °C for 8 h.

### Photocatalytic Membrane Preparation

To fabricate the SSPM, a piece of clean PVDF membrane was soaked in the mixed solution of DMF and ethanol (DMF: ethanol = 1: 1 v/v) for 2 min to soften the PVDF fibers. Then, certain amount of catalyst (2 mg unless otherwise specified) was dispersed in 20 mL of the mixed solution (DMF: ethanol = 1: 1 v/v). The suspension solution was filtrated through the above-softened PVDF membrane by vacuum filtration to allow catalyst deposition. Next, the membrane was placed into a fume hood overnight to evaporate the residual DMF and ultimately obtain the SSPM.

For fabrication of the MEPM, the PVDF powder and particulate catalyst were mixed (catalyst: PVDF = 7: 93 w/w) and suspended in the DMF solution. The as-prepared solution was cast on a glass plate using automatic coating machine and casting knife and then immersed in deionized for phase-inversion to form the MEPM. In addition, a surface-unbounded photocatalytic membrane (SUPM), with the catalyst loosely loaded on the membrane surface, was also fabricated by a simple vacuum filtration method. Specific amount of particulate catalysts was suspended in 20 mL ethanol and then filtrated through the PVDF membrane. Then, the membrane was placed in a fume hood overnight to obtain the SUPM. The catalyst loading amount of membrane was calculated based on the catalyst weight and loaded area according to Eq. (1):

1 $$\begin{array}{c}Loaded\, amount= \frac{\text{catalyst weight}}{\text{effeective loaded area on membrane}}\end{array}$$

### Photocatalytic Experiments

#### ***H***_***2***_***O***_***2***_*** Photosynthesis Experiment***

The H_2_O_2_ photosynthesis experiment was performed in a beaker, fed with 30 mL DI water and purged with O_2_ for 30 min to ensure O_2_–saturation. Then, the photocatalytic membrane was immersed in the water solution, and light irradiation was applied to initiate the reaction. A 300-W Xenon lamp (Beijing Pulinsaisi Technology Co., Ltd.)-equipped filter of AM 1.5G was used as the light source. The light wavelength was from 300 to 1600 nm, and the applied light intensity was 100 mW cm^−2^. The reaction temperature was controlled at 10 °C. The water samples were collected from the beaker every 20 min during the reaction. For the cyclic experiment, the photocatalytic membrane was cleaned by soaking it in DI water for three times without rinsing, thus avoiding the possible detachment of catalyst from SUPM during non-reaction periods. The H_2_O_2_ concentration was measured by titanium sulfate (Ti(SO_4_)_2_) colorimetry [34]. Specifically, 0.5 mL sample and 0.5 mL Ti(SO_4_)_2_ solution were mixed, and the absorbance at 410 nm was measured by ultraviolet spectrometry.

#### Micropollutants’ Degradation Experiment

The pollutant photodegradation experiment was conducted in a beaker. A low-pressure Hg UV lamp (254 nm wavelength) was used as the light source. The UV fluence was calculated to be 1.08 mW cm^−2^ based on the KI/KIO_3_ actinometer measurement [35]. TC and BPA, which are ubiquitous in aquatic environment, were selected as model pollutants to evaluate the photocatalytic decontamination performance following the reported procedures [36–38]. Prior to the degradation test, the photocatalytic membrane was immersed 30 mL water solution (containing 5 mg L^−1^ pollutant) and mixed for 15 min in the dark to reach absorption equilibrium. During the photodegradation experiment, the samples were collected regularly for concentration measurement. The TC concentration was measured by a UV spectrometer at 357 nm. The BPA concentration was measured by high-performance liquid chromatography at 273 nm with methanol and pure water mobile phase (70:30 v/v). The formed intermediates during pollutant degradation were detected by UPLC-mass spectrometry (Waters XEVO G2-XS, QTOF) in a negative scan mode of the electron spray ionization source. The mobile phase for BPA measurement was a mixture of acetonitrile/ammonium hydroxide (5 mM) (50:50, v/v), and the mass calibration range was between 50 and 1000 Da with the resolution above 24,000. The mobile phase for TC measurement was a mixture of formic acid solution (0.1%, m/m) and acetonitrile (90:10, v/v), and the mass calibration range was between 50 and 1000 Da with the resolution above 24,000.

## Results and Discussion

### Characteristics of the Surface Self-Bounded Photocatalytic Membrane

**Fig. 1:**
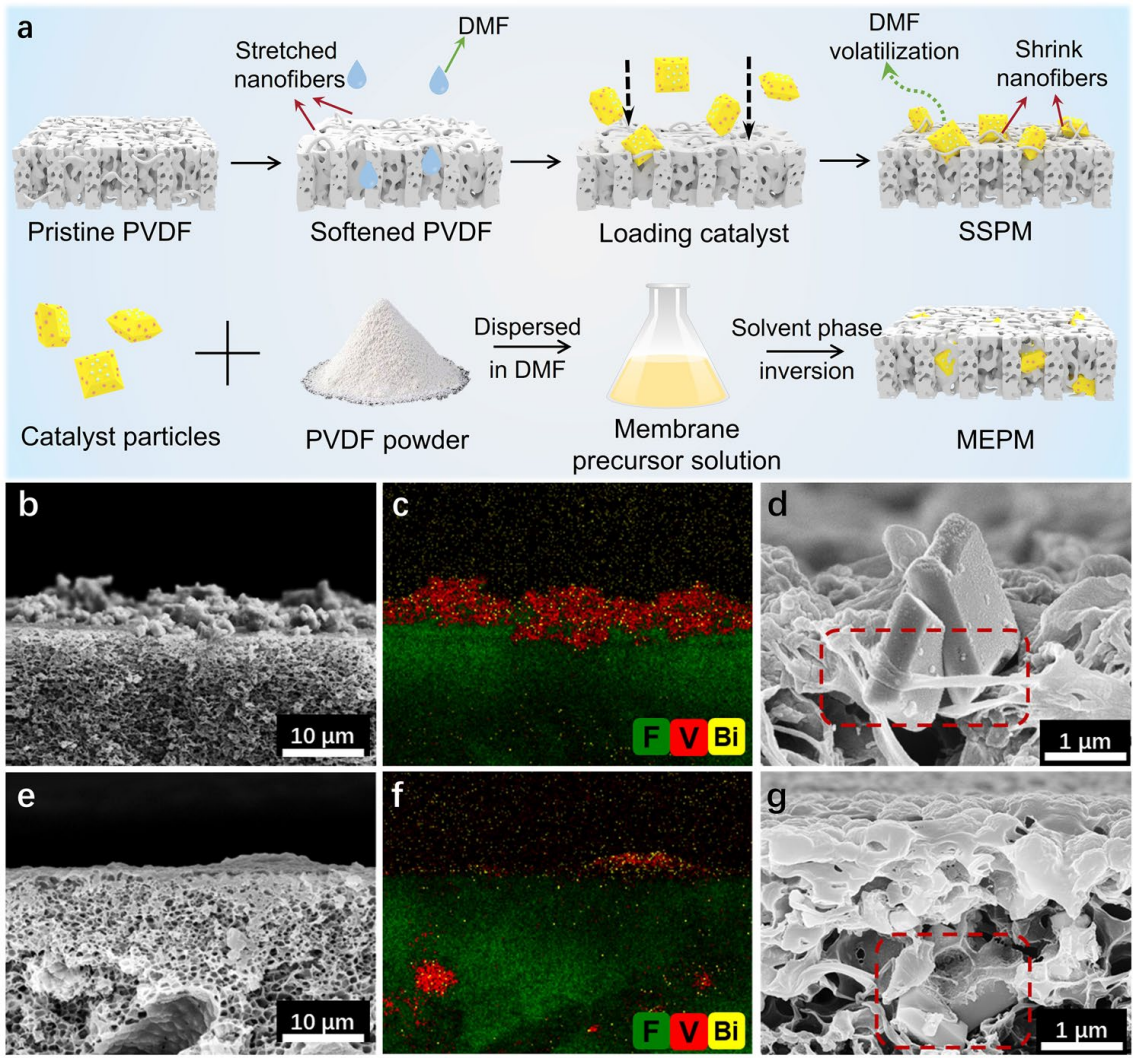
**a** Procedures for fabrication of SSPM and MEPM; The SEM image, EDS mapping, and high-resolution SEM image of membrane cross-section for **c-e** SSPM and **f–h** MEPM. The red dotted box shows the position of photocatalyst

The SSPM and MEPM, both loaded with faceted CoO_x_/Mo:BiVO_4_/Pd as the photocatalyst, were fabricated following the procedures illustrated in Fig. 1a. The composition and structure of the photocatalyst were verified by the X-ray diffraction (XRD) and X-ray photoelectron spectra (XPS) (Figs. S1 and S2). The scanning electron microscopic (SEM) images and energy-dispersive X-ray spectroscopy (EDS) mapping of the SSPM cross-section (Figs. 1b, c and S3a) confirm the deposition of a dense BiVO_4_ photocatalyst layer on the membrane surface, indicating that they were well retained by the micropores and dispersed on the PVDF surface. A closer observation by high-resolution SEM revealed that the surface-loaded photocatalyst particles were tightly strapped by the PVDF nanofibers, but the catalyst surfaces were sufficiently exposed (Figs. 1d and S3b, c). Notably, the membrane structure of SSPM remained intact, although the membrane shape and internal surface area changed slightly relative to the pristine PVDF membrane (Fig. S4 and Table S1). In contrast, for the MEPM the majority of photocatalysts was embedded within the PVDF matrix rather than on the surface (Fig. 1e-g). Besides, the surface electric potential of SSPM changed from -11.4 to -27.6 mV when raising the pH from 4.0 to 9.0 (Fig. S5a). The surface water contact angle of SSPM was 120.9°, indicating that it had a hydrophobic surface (Fig. S5b).

The SUPM with a loosely loaded photocatalyst on the surface (obtained by vacuum filtration without DMF treatment) was also fabricated for comparison (Fig. S6a). The characterization results confirm that the photocatalyst particles had relatively loose contact with the membrane surface (Fig. S6b), making them vulnerable to detachment under hydraulic water flow condition, as evidenced by the 84.6% loss of catalyst after 30-min crossflow operation with a flow rate of 350 mL min^−1^ (Fig. S7). In comparison, the SSPM exhibited a negligible catalyst loss (~ 5.4%), confirming its high mechanical stability. Moreover, the photocatalysts collected from the SSPM exhibited similar morphology and structure to the pristine ones (Fig. S1), indicating that they were physically immobilized without altering the inherent properties. These favorable features rendered the SSPM high efficiency and robustness for continuous-flow water treatment.

### H_2_O_2_ Photosynthesis Performance of Photocatalytic Membranes

**Fig. 2:**
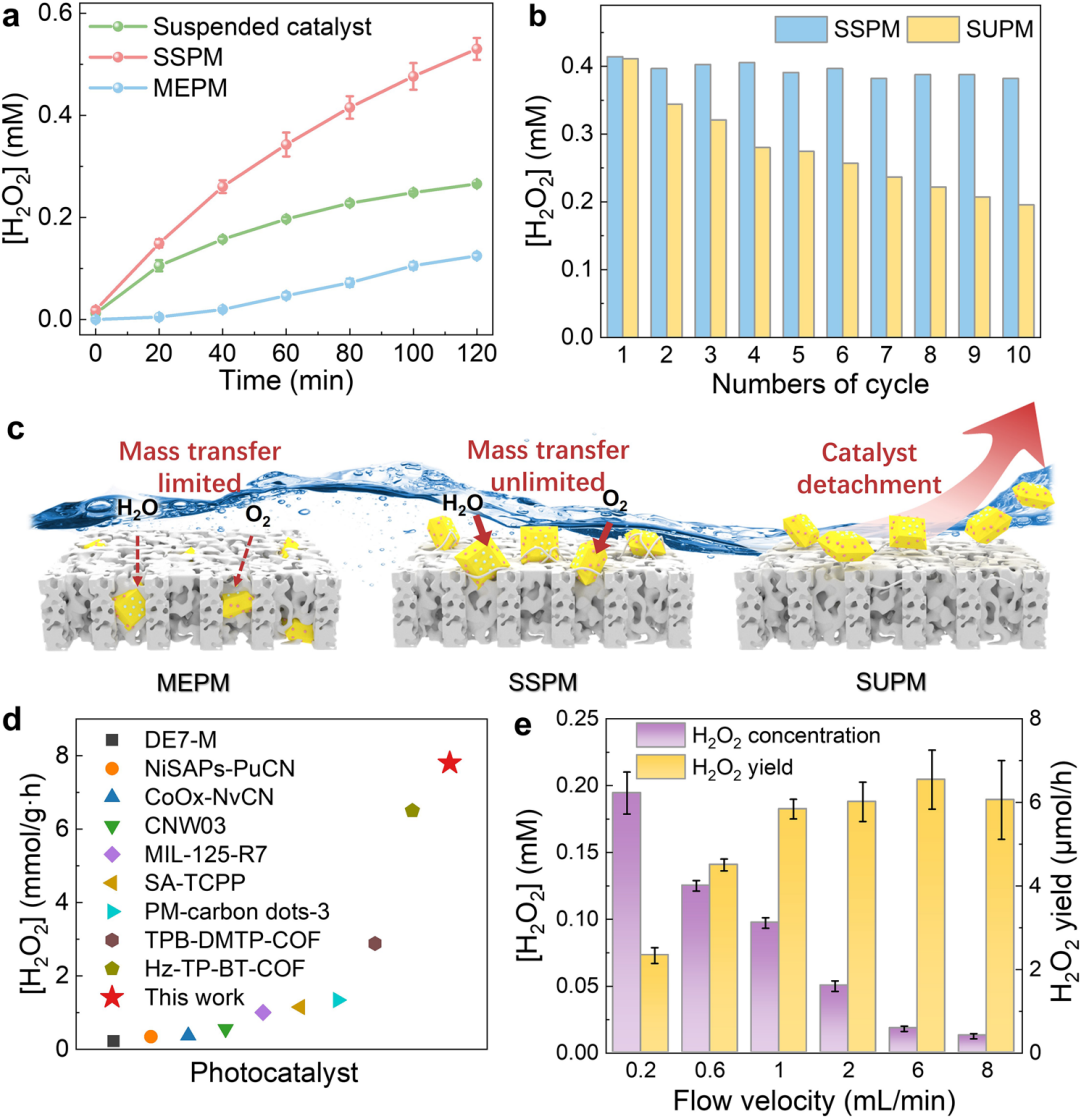
**a** Photocatalytic H_2_O_2_ generation by different membranes in a typical batch operation and **b** during 10 operating cycles. **c** Schematic diagram of the membrane under flow-by operation illustrating the different reactant transfer conditions and catalyst stability. **d** Reactivity in comparison with other photocatalysts in the literature. **e** H_2_O_2_ production during continuous-flow operation with different flow rates.

The H_2_O_2_ photosynthesis activities of the different photocatalytic membranes, all loaded with 1.59 g m^−2^ of photocatalyst, were first evaluated by batch experiment. Here, simulated sunlight was adopted as the light source to facilitate quantification of H_2_O_2_ production, otherwise under UV-light H_2_O_2_ photolysis would easily occur. The SSPM immersed in water exhibited much higher activity than the MEPM for H_2_O_2_ generation (0.53 vs. 0.12 mM within 2 h) (Fig. 2a). In addition, it also exhibited a higher H_2_O_2_ production rate (7700 μmol g^−1^ h^−1^) than the majority of the reported photocatalyst (220 ~ 6500) μmol g^−1^ h^−1^ (Fig. 2d and Table S2). Here, the superior photocatalytic activity of the SSPM should be mainly ascribed to the highly exposure degree of its surface-bounded photocatalyst, which favors the light adsorption and easy access of O_2_ and H_2_O for the photocatalytic reaction (Figs. 2c and S8). In contrast, the slow diffusion of O_2_ to the embedded photocatalysts and local accumulation of the generated H_2_O_2_ severely restricted the MEPM performance.

In addition, the SSPM with firmly bounded catalyst maintained high photocatalytic activity during 20 cyclic runs without obvious performance decline (Figs. 2b and S9), indicating its superior stability during long-time operation. This is also supported by the SEM image that shows abundant remaining particulate catalyst on the membrane surface after reaction (Fig. S10). In comparison, the SUPM (with loosely immobilized catalyst) exhibited 54.7% activity decay after 10 running cycles, while the membrane-free suspension system (with powder catalyst) exhibited negligible activity after 4 cycles (Fig. S11), possibly due to loss or agglomeration of the suspended particulate catalyst.

To evaluate the potential of the different photocatalyst membranes for practical application, their performances in a membrane cell under continuous-flow operation were also evaluated (Fig. S12). Here, the membrane only served as a photocatalyst carrier and was operated under flow-by mode: Water flew through the channels above the membrane instead of passing through the membrane matrix since no transmembrane pressure was applied. As expected, the SSPM also stood out as the best one, exhibiting 19-folds higher H_2_O_2_ production rate than the MEPM (Fig. S13). Notably, the overall H_2_O_2_ yield of the SSPM could be facilely tuned. The overall H_2_O_2_ production rate increased from 2.33 to 6.05 μmol h^−1^ when increasing the water flow from 0.2 to 8 mL min^−1^ (Fig. 2e), due to the enhanced interfacial mass transfer under faster flow conditions.

### Reactant Availability and H_2_O_2_ Diffusion of Different Membranes

**Fig. 3:**
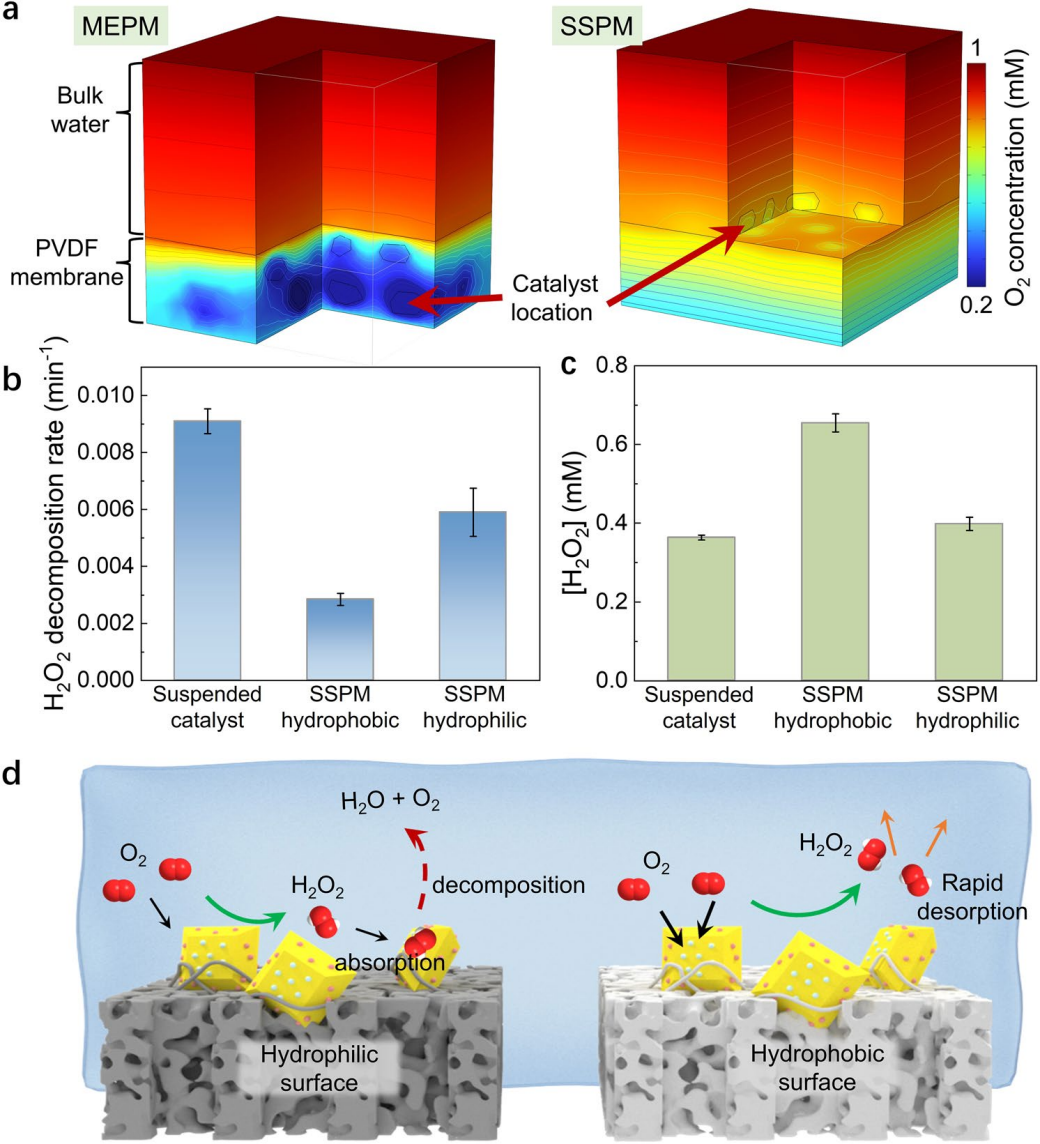
**a** Modeling result of oxygen distribution in MEPS and SSPM, with bulk O_2_ concentration of 1 mM and surface oxygen consumption rate of 0.01 mmol s^−1^ (resembling the real reaction condition). **b** H_2_O_2_ decomposition rate and **c** accumulative H_2_O_2_ production in different photocatalytic systems (Suspended catalyst, SSPM hydrophobic and SSPM hydrophilic). **d** Schematic diagram of H_2_O_2_ generation, diffusion and decomposition processes of SSPM with hydrophilic (left) and hydrophobic (right) surface

Efficient diffusion of dissolved O_2_ is critical for the H_2_O_2_ photosynthesis in our photocatalytic membrane system (O_2_ + 2e^−^ + 2H^+^  → H_2_O_2_) (Fig. S14). The result of Multi-Physics Simulation shows obviously accelerated O_2_ diffusion and increased O_2_ accessibility for SSPM than MEPM (Figs. 3a, S15, and S16). The MEPM exhibits a sharp decline in O_2_ concentration across the PVDF matrix due to the sluggish diffusion rate, thus resulting in severe O_2_ deficiency for the embedded photocatalysts. In contrast, higher-concentration O_2_ was available for the surface-exposed photocatalyst of SSPM, rendering it a high H_2_O_2_ production kinetics.

Apart from the improved O_2_ availability, the improved diffusion and hence less decomposition of the generated H_2_O_2_ by SSPM also contributed to its high H_2_O_2_ productivity. In the MEPM, the diffusion of H_2_O_2_ from the matrix-embedded photocatalyst to the bulk water was restricted; thus, the accumulated H_2_O_2_ would undergo rapid decomposition over the photocatalysts (H_2_O_2_ + 2 h^+^  → 2H^+^  + O_2_) [39, 40]. In comparison, the generated H_2_O_2_ can easily diffuse away from the catalyst on SSPM surface, rendering it even higher H_2_O_2_ production rate than in the suspended catalyst system (Fig. 2a) due to increased H_2_O_2_ desorption/diffusion from the hydrophobic membrane surface [41–43] and hence less H_2_O_2_ decomposition (Figs. 3b and S17). This was further supported by the fact that shifting from hydrophobic to hydrophilic membrane led to drastically accelerated H_2_O_2_ decomposition over the catalytic membrane (Fig. S5b). Overall, the efficient reactant diffusion and less H_2_O_2_ decomposition for the exposed photocatalyst rendered the SSPM superior activity for H_2_O_2_ photosynthesis (Fig. 3c, d).

### Pollutant Photodegradation Performances and Pathways

**Fig. 4:**
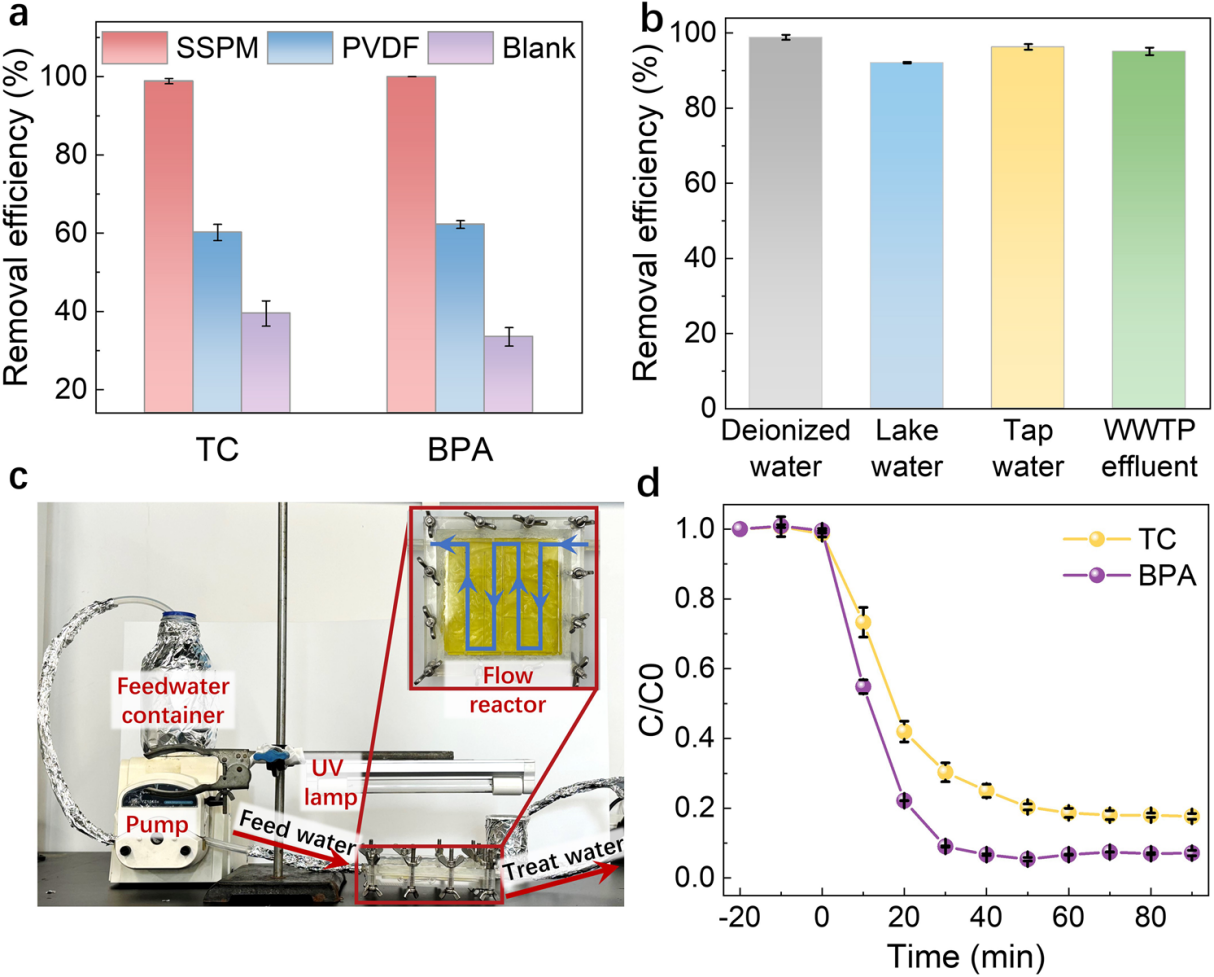
**a** Pollutant removal ratios of the SSPM under UV light; **b** TC removal ratios of the SSPM for treatment of real water samples; **c** Photograph of experimental setup and **d** pollutant performance of the SSPM-based photocatalytic membrane reactor setup for continuous-flow water treatment. Reaction conditions of batch experiment: pollutant concentration = 10 mg L^−1^; catalyst loading amount of membrane = 3.98 g m^−2^; UV fluence = 1.08 mW cm^−2^, reaction time was 60 min; Reaction conditions of continuous-flow reactor: pollutant concentration = 5 mg L^−1^; catalyst loading amount of membrane = 3.98 g m^−2^; UV fluence = 1.08 mW cm^−2^, the hydraulic retention time was 50 min (flow rate = 0.2 mL min.^−1^)

The high H_2_O_2_ photosynthesis activity and stability of the SSPM make it highly adaptable to the widely adopted UV/H_2_O_2_ processe for water treatment application. We first optimized the photocatalytic performance of the SSPM by batch experiment (Fig. S18). The membrane with optimal catalyst loading of 3.98 g m^−2^ and DMF/ethanol ratio of 1:1 (v/v) was adopted for the subsequent experiments. Impressively, the SSPM achieved complete pollutant removal within 60 min under UV irradiation (Fig. 4a), exhibiting tenfold higher reaction kinetics than that of the UV alone group (Fig. S19). Accordingly, the TOC removal efficiencies were 66.6% and 71.6% for TC and BPA degradation, respectively (Fig. S20). In addition, it exhibited negligible pollutant removal in the dark (Fig. S21), indicating a photodegradation-dominated decontamination process.

The reactive oxygen species (ROS) responsible for pollutant degradation in the UV photocatalytic system was further identified by adding different inhibitors or scavengers: tertiary butanol (TBA) for ·OH, ammonium oxalate (AO) for holes and L-histidine (L-his) for ^1^O_2_) (Fig. S22). The markedly inhibited BPA degradation by N_2_ purging, which prohibited the generation, confirms a synergistic action between the generated H_2_O_2_ and UV for augmenting ROS generation and pollutant degradation. Significant suppression was also caused by TBA and L-his, indicating the generation of abundant ·OH and ^1^O_2_ in the reaction system. Here, the ·OH should be generated by H_2_O_2_ photolysis (H_2_O_2_ + *hv* → 2·OH) and ^1^O_2_ was generated by the ·OH disproportionation (4·OH → ^1^O_2_ + 2H_2_O) [44].

The efficient decomposition of BPA and TC was further validated by liquid chromatograph-mass spectroscopy (LC–MS). According to the values of m/z and chemical structure of intermediate products identified (Fig. S23), the BPA degradation in the photocatalytic system should proceed by two pathways (Fig. S24a). In the first way, the BPA molecule undergoes C–C bond cleavage to form P1, which is further transformed into P2 and P3 through the hydroxylation and oxidization reaction [45]. In the second way, BPA is degraded to P4 and P5 by ·OH attacking; P5 is further transformed into P6 and P7 through dehydroxylation and carbonylation [46], followed by further bond cleavage to form P8, P9 and P10 or mineralization into CO_2_ and H_2_O. The TC degradation also proceeds by two degradation ways (Fig. S24b). In the first way, TC is attacked by radicals and undergoes demethylation to yield TP1, which further lost the methyl group to form TP2 [47]. In pathway 2, TC undergoes dehydration and N-demethylation to form TP3 and TP4 [48], which is further oxidized into smaller molecules (TP6, TP7, TP8 and TP9) through the cleavage of additional functional groups, intermolecular rearrangement and ring-opening reactions, eventually being mineralized to CO_2_ and H_2_O.

### Potential for Water Treatment Application

Attributed to the radical–nonradical mixed pathway, the SSPM exhibited superior environmental robustness, remaining over 95% of the degradation activity in the presence of Cl^−^, NO_3_^−^ and humic acid, and 80% activity under exposure to SO_4_^2−^ and HCO_3_^−^ (Fig. S25). Here, the slight suppression by SO_4_^2−^ and HCO_3_^−^was likely due to SO_4_^2−^ competitive absorption on catalyst active site and ·OH scavenging by HCO_3_^−^ [49, 50]. The SSPM also demonstrated superior performance for treatment of real water samples, including lake water, tap water, and secondary effluent from wastewater treatment plant, all achieving > 95% TC removal within 60 min (Fig. 4b).

 To facilitate continuous-flow operation for practical water treatment, we further evaluated the SSPM performance under flow-by mode as mentioned above (Fig. 4c). As expected, the system steadily maintained 82% and 93% removal rates for TC and BPA, respectively (Fig. 4d). The superior stability of the SSPM is not only ascribed to the chemical stability of the inorganic catalyst and the PVDF membrane, which are resistant to the oxidative environment (Fig. S26), but also benefited from the unique catalyst immobilization method adopted in this study. In addition, the generated reactive oxygen species can in situ eliminate the membrane foulants (Fig. S27), thus ensuring a long-term stable operation of the SSPM system. All these results suggest that the SSPM could be readily integrated into the UV/H_2_O_2_ processes to facilitate practical water treatment application.

Apart from catalytic activity, economic cost is also an important consideration for evaluating the application potential of a technology. Therefore, we approximately estimated the fabrication costs (including the materials and energy consumption) of the photocatalytic membranes (detailed in the supplementary information Tables S3-S5). The SSPM and MEPM with different catalyst loadings were fabricated to ensure similar H_2_O_2_ photosynthesis performances. A comparison of the fabrication cost shows that the SSPM can achieve 39.3% cost saving relative to MEPM (9.948 vs. 16.392 USD m^−2^) (Fig. S28). Another economic advantage of the SSPM lies in its easy storage in dry state (Fig. S29), in sharp contrast to deactivation of the MEPM after dry storage.

Notably, although we only showcase the construction of SSPM by immobilizing CoO_x_/BiVO_4_/Pd inorganic catalyst on a hydrophobic PVDF membrane, this strategy may be readily extended to other photocatalytic membranes with diverse catalyst types and membrane properties. For example, the particulate catalyst was also successfully self-bounded to the surface of a hydrophilic PVDF membrane, exhibiting similar decontamination performance to that with hydrophobic surface (Fig. S30). This is different from the H_2_O_2_ photosynthesis process that depends strongly on the membrane surface property, because the generated H_2_O_2_ was rapidly activated on membrane surface regardless of its hydrophilicity. In addition, the organic photocatalyst of RF523, a spherical particulate catalyst with diameter of 800 nm, could also be immobilized on PVDF membrane to form a SSPM, achieving tenfold higher H_2_O_2_ photosynthesis rate than the MEPS (Fig. S31).

Lastly, apart from the crossflow operating mode adopted for the photocatalytic membrane application, the SSPM with hydrophilic surfaces may also be constructed to facilitate flow-through filtration application in the future. In addition, suitable photoreactors should be developed for better integration of the SSPM into the practical UV/H_2_O_2_ water treatment processes.

## Conclusion

A unique SSPM was fabricated, and its superior performance for photocatalytic water decontamination was demonstrated. The immobilization strategy adopted here, i.e., chemically softening the PVDF fibers for firmly strapping the photocatalyst particles on the membrane surface, can overcome the challenge of the conventional immobilization approaches that typically compromise the catalytic activity. Benefited from the sufficient exposure of the photocatalysts on membrane surface to improve O_2_ accessibility and H_2_O_2_ diffusion, the SSPM exhibited superior photocatalytic activity, achieving a 4.2-fold higher rate of photosynthesis and tenfold higher kinetics of pollutant degradation than the MEPM under UV irradiation. Our work lays a key basis for advancing photocatalytic technology toward sustainable water purification applications and may offer valuable implications for improved design of various catalytic membranes.

## Supplementary Information

Below is the link to the electronic supplementary material.

Supplementary file1 (DOCX 3605 KB)
