# Supplementary material for: Binder-Free Immobilization of Photocatalyst on Membrane Surface for Efficient Photocatalytic H2O2 Production and Water Decontamination
Source: Nanomicro Lett. 2025 Jun 18;17:301. doi: 10.1007/s40820-025-01822-0 (PMC12176716; doi:10.1007/s40820-025-01822-0)
Supplement: Supplementary file 1 — Supplementary file1 (DOCX 3605 KB) [file 40820_2025_1822_MOESM1_ESM.docx]

Supporting Information for

**Binder-Free Immobilization of Photocatalyst on Membrane Surface for Efficient Photocatalytic H_2_O_2_ Production and Water Decontamination**

Zhen-Yu Hu ^1,2,4^, Tian Liu ^1,2,3^*,Yu-Ru Yang^1,2^, Alicia Kyoungjin An^4,5,^*, Kim Meow LIEW ^2,5^, Wen-Wei Li ^1,2,^*

^1^ State Key Laboratory of Advanced Environmental Technology, Department of Environmental Science and Engineering, University of Science and Technology of China, Hefei 230026, P. R. China

^2^ SEEM Innovation Center, Suzhou Institute for Advanced Research, University of Science & Technology of China, Suzhou 215123, P. R. China

^3^ School of Resources and Environmental Engineering, Hefei University of Technology, Hefei 230009, P. R. China

^4^ School of Energy and Environmental, City University of Hong Kong, Hong Kong SAR 999077, P. R. China

^5^ Department of Chemical and Biological Engineering, Hong Kong University of Science and Technology, Hong Kong SAR, P. R. China

^6^ Department of Architecture and Civil Engineering, City University of Hong Kong, Hong Kong SAR 999077, P. R. China

***** Corresponding authors. E-mail: [liutian123@ustc.edu.cn](mailto:liutian123@ustc.edu.cn) (Tian Liu); [alicia.kjan@ust.hk](mailto:alicia.kjan@ust.hk) (Alicia Kyoungjin An); [wwli@ustc.edu.cn](mailto:wwli@ustc.edu.cn) (Wen-Wei Li)

**S1 Multi- Physics Simulation Method**

The oxygen concentration distribution over the SSPM and MEPM were analyzed using COMSOL Multiphysics 6.2. The geometric model was constructed as a 100×100×100 um cubic domain, with the membrane represented as a porous medium. Catalyst particles were modeled as octahedral structures (1-2 um in diameter), randomly deposited on the membrane outer surface for SSPM or uniformly dispersed within the membrane matrix for MEPM. A physics-controlled mesh with boundary layer refinement was applied near catalysts and interfaces to resolve steep concentration gradients. The oxygen distribution in a solution was simulated using the Dilute Species Transport module in COMSOL. The governing equation for dilute species transport is:

∇·J_i_ + u·∇c_i_ = R_i_, where J_i_ = -D_i_∇c_i_

were J_i_ refers to species flux, u is fluid velocity field, c_i_ is the concentration of the species, R_i_ is Reaction term; D_i_ is the Diffusion coefficient. The diffusion term is calculated using Fick’s first law, where the flux (J_i_) divided by the concentration gradient (∇c_i_) equals the diffusion coefficient (D_i_).

**Table S1** BET surface areas of the pristine PVDF, SSPM and SUPM

|  | Pristine PVDF | SSPM | SUPM |
| --- | --- | --- | --- |
| Surface area (m^2^/g) | 18.1 | 6.47 | 17.9 |

**Table S2** Summary of recently reported materials for H_2_O_2_ photosynthesis

| **Photocatalyst** | **Reaction solution** | **Light source** | **H_2_O_2_ yield**  **(μmol g^-1^ h^-1^)** | **Refs.** |
| --- | --- | --- | --- | --- |
| DE7-M | Pure water | λ ≥ 420 nm | 221.6 | **[S**1] |
| NiSAPs-PuCN | Pure water | λ ≥ 420 nm | 342.2 | [S2] |
| CoOx-NvCN | Pure water | AM 1.5G | 367.8 | [S3] |
| CNW03 | Pure water | λ ≥ 420 nm | 556 | [S4] |
| MIL-125-R7 | Benzyl alcohol solution | λ ≥ 420 nm | 1000 | [S5] |
| SA-TCPP | Pure water | λ ≥ 420 nm | 1150 | [S6] |
| PM-carbon dots-3 | Pure water | λ ≥ 420 nm | 1340 | [S7] |
| TPB-DMTP-COF | Pure water with tris-phase reactor | λ ≥ 420 nm | 2882 | [S8] |
| Hz-TP-BT-COF | Pure water | λ ≥ 420 nm | 6500 | [S9] |
| This work | Pure water | AM 1.5G | 7700 | - |

**Table S3** The cost of raw materials for fabricating 1 g CoO_x_/Mo:BiVO_4_/Pd

| **Reagent** | **Dosage**  **(g or mL)** | **Unit price**  **(USD/g or USD/mL)** | **Total price**  **(USD)** |
| --- | --- | --- | --- |
| K_2_CO_3_ | 0.349 | 0.009 | 0.003 |
| V_2_O_5_ | 0.758 | 0.134 | 0.101 |
| K_2_MoO_4_ | 0.001 | 0.825 | 0.001 |
| Bi(NO_3_)_3_ | 1.617 | 0.005 | 0.001 |
| Ethanol | 9.667 | 0.011 | 0.111 |
| Co(NO_3_)_2_ | 0.003 | 0.042 | 0.000127 |
| NaPdCl_4_ | 0.004 | 54.319 | 0.217 |
| NaIO_3_ | 0.5 | 0.265 | 0.133 |
| Total | - | - | 0.574 |

**Table S4** The energy consumption for fabricating 1 g catalyst or 1 m^2^ catalytic sheet

|  | **Program** | **Energy consumption (kW·h)** | **Energy price (USD/kW·h)** | **Cost of energy consumption (USD)** |
| --- | --- | --- | --- | --- |
| Preparing catalyst | Water bath | 5.95 | 0.085 | 0.506 |
|  | Muffle furnace | 6.933 |  | 0.589 |
|  | Xenon lamp | 0.7 |  | 0.06 |
|  | Total | 13.583 |  | 1.155 |
|  |  |  |  |  |
| Preparing SSPM | Water bath | 3.15 | 0.085 | 0.268 |
|  | Membrane coating machine | 0.208 |  | 0.018 |
|  | Vacuum filtration | 0.09 |  | 0.008 |
|  | Total | 3.448 |  | 0.294 |
|  |  |  |  |  |
| Preparing MEPM | Water bath | 3.15 | 0.085 | 0.268 |
|  | Membrane coating machine | 0.208 |  | 0.018 |
|  | Total | 3.358 |  | 0.286 |

**Table S5** The cost of material and energy consumption for preparing 1 m^2^ photocatalytic membrane

| **Sheet type** | **Reagent** | | **Dosage**  **(g, mL or kW·h)** | **Unit price**  **(USD/g, USD/mL or kW·h)** | **Total price**  **(USD)** |
| --- | --- | --- | --- | --- | --- |
| SSPM | CoO_x_/Mo:BiVO_4_/Pd | | 1.861 | 0.574 | 1.068 |
|  | PVDF | | 30.707 | 0.154 | 4.729 |
|  | DMF | | 193.552 | 0.008 | 1.593 |
|  | Ethanol | | 10 | 0.011 | 0.115 |
|  | Energy consumption | for catalyst | 25.277 | 0.085 | 2.149 |
|  |  | for SSPM | 3.448 | 0.085 | 0.294 |
|  | Total | | - | - | 9.948 |
| MEPM | CoO_x_/Mo:BiVO_4_/Pd | | 5.864 | 0.574 | 3.365 |
|  | PVDF | | 29.387 | 0.154 | 4.526 |
|  | DMF | | 175.658 | 0.008 | 1.446 |
|  | Ethanol | | 0 | 0.011 | 0 |
|  | Energy consumption | for catalyst | 79.65 | 0.085 | 6.77 |
|  |  | for MEPM | 3.358 | 0.085 | 0.286 |
|  | Total | | - | - | 16.392 |

**Supplementary Figures**


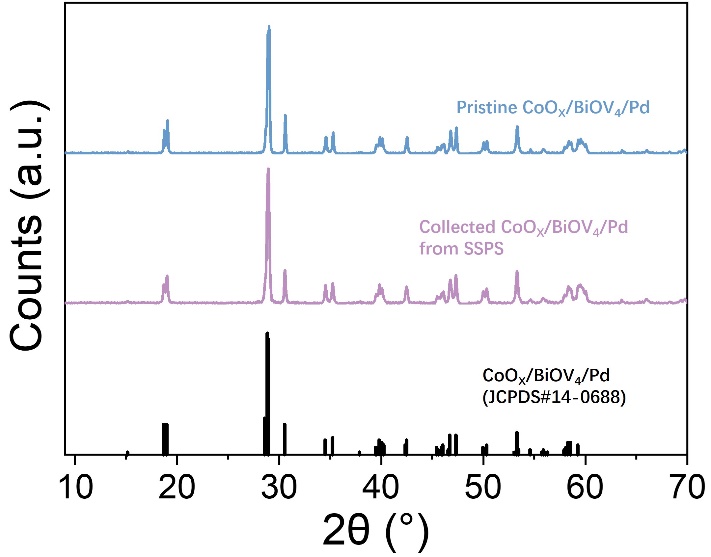


**Fig. S1** XRD pattern of the pristine CoO*_x_*/Mo:BiVO_4_/Pd particles and those collected from SSPM. The XRD pattern is in good agreement with JCPDS standard card #14-0688, corresponding to monoclinic sheelite BiVO_4_


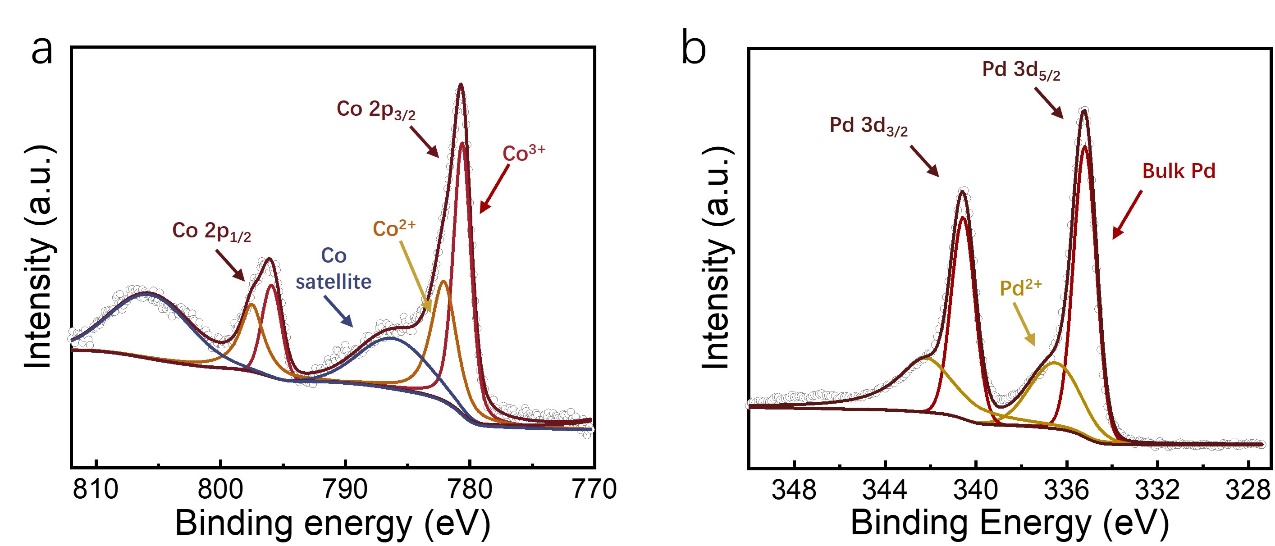


**Fig. S2** (**a**) Co 2p XPS spectra of CoO*_x_*/Mo:BiVO_4_. (**b**) Pd 3d XPS spectra of Mo:BiVO_4_/Pd. The area of Co 2p_3/2_ peak is twice that of Co 2p_1/2_ peak and the binding energies of Co^3+^ and Co^2+^ are 780.6 and 781.6 eV, respectively. The area of Pd 3p_5/2_ peak is 1.5 times that of Pd 2p_3/2_ peak and the binding energies of bulk Pd and Pd^2+^ are 335.1 and 337.0 eV, respectively


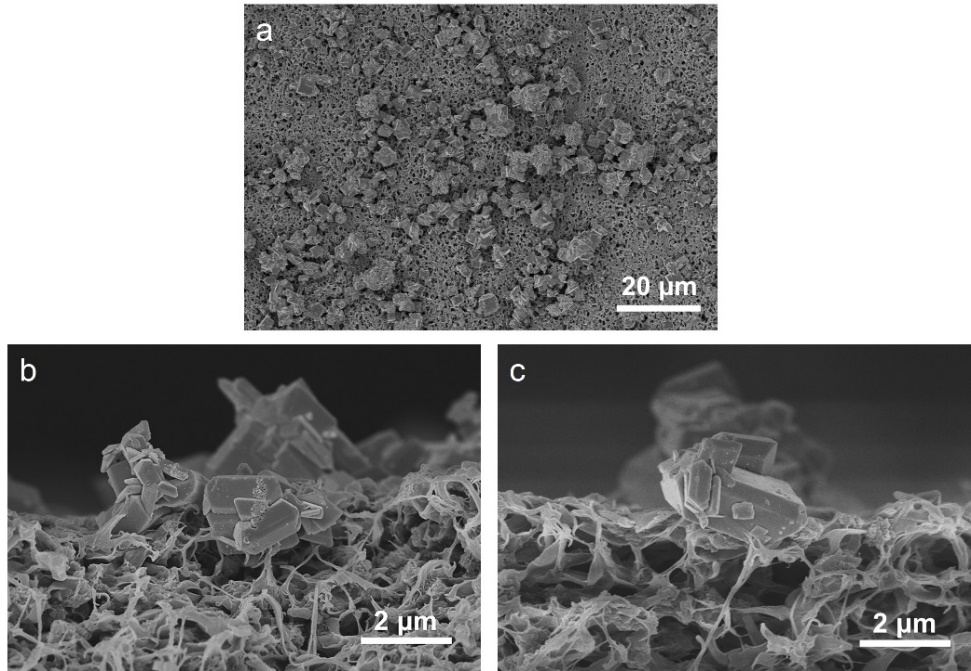


**Fig. S3** (**a**) SEM images of the distribution state of particulate catalyst on the SSPM surface. (**b**) and (**c**) the high resolution of SEM images of particulate catalyst bounded by PVDF fibers


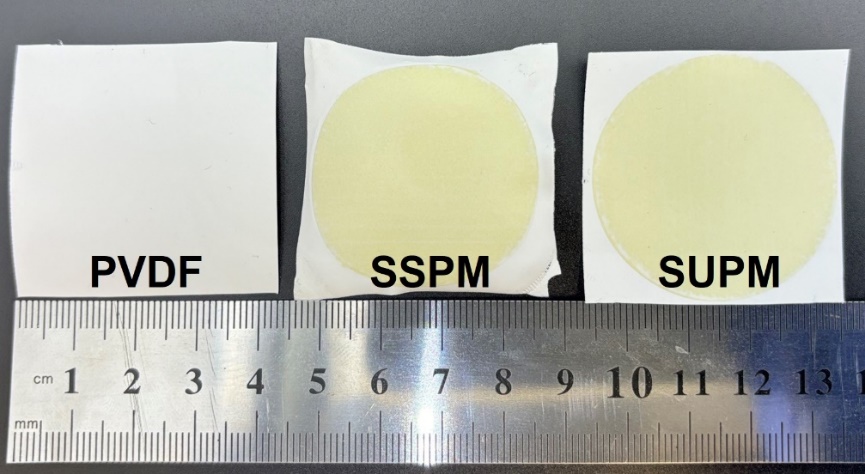


**Fig. S4** The shape comparison of pristine PVDF (left), SSPM (middle) and SUPM (right)


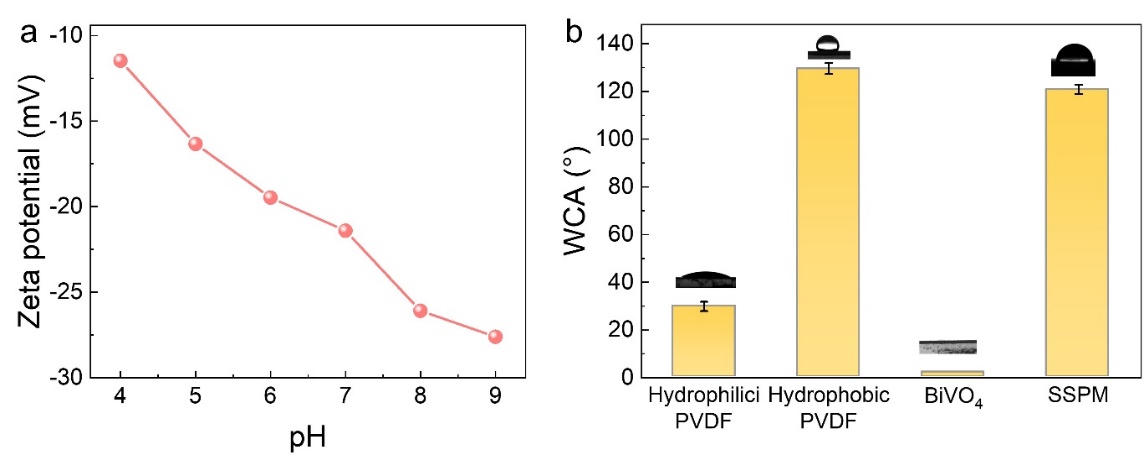


**Fig. S5** (**a**) The surface potential of SSPM under different pH solutions. (**b**) The water contact angle of hydrophilic PVDF, hydrophobic PVDF, catalyst powder and SSPM


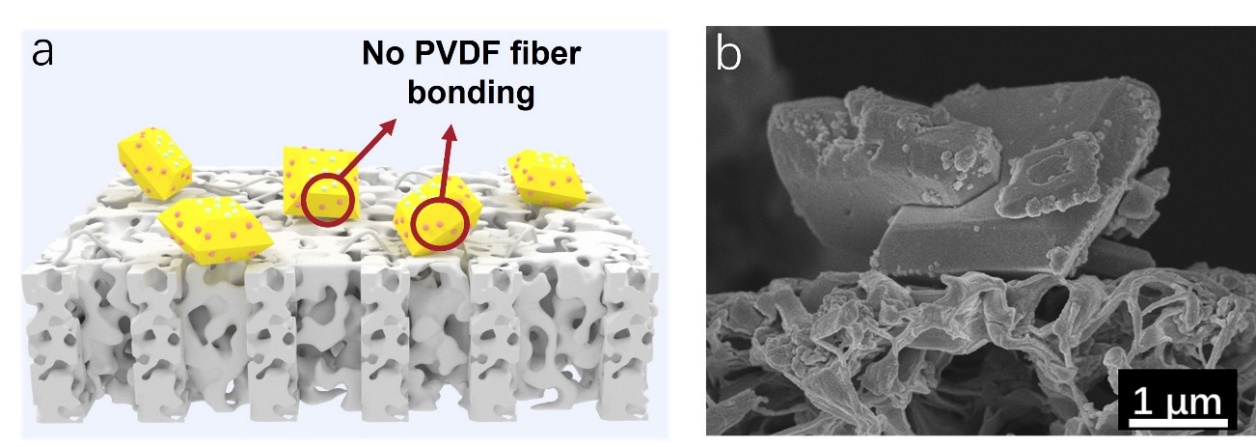


**Fig. S6** (**a**) Schematic diagram of the SUPM. (**b**) SEM image of SUPM showing lack of catalyst binding to the PVDF fibers

**
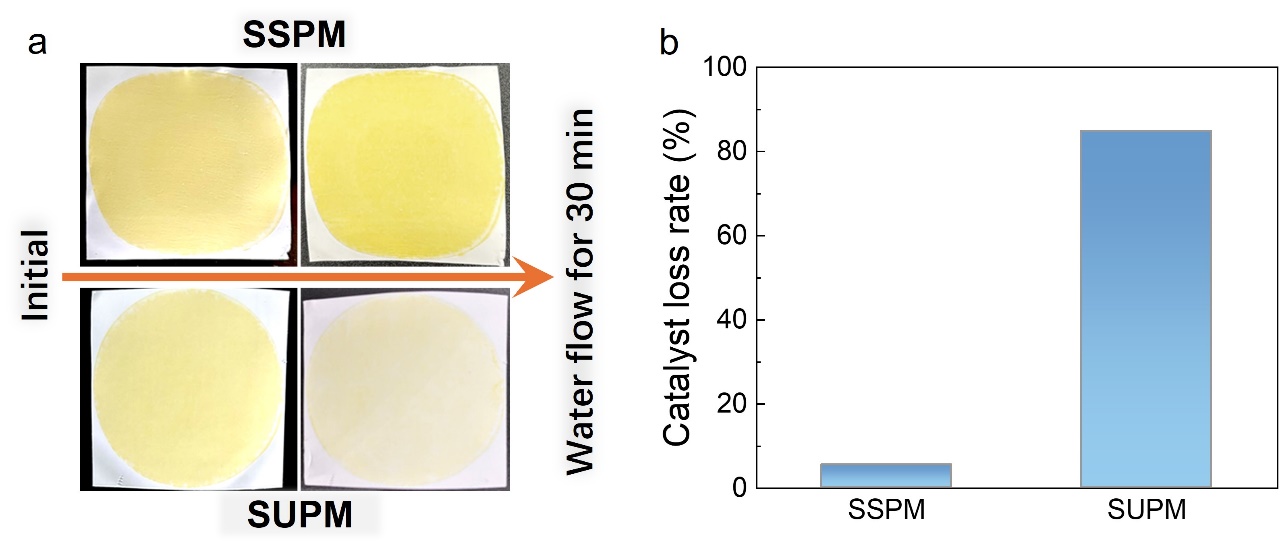
**

**Fig. S7** (**a**) Photographs of SSPM and SUPM before and after cross flowing. (**b**) Catalyst loss rate of SSPM and SUPM after water cross flowing


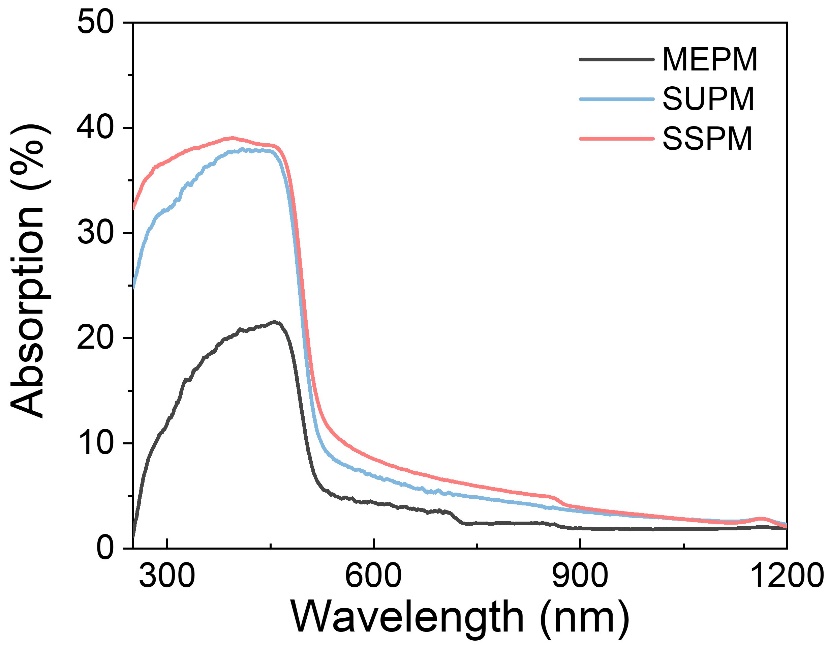


**Fig. S8** UV-vis absorption spectra (250 -1200 nm) of different photocatalytic membranes


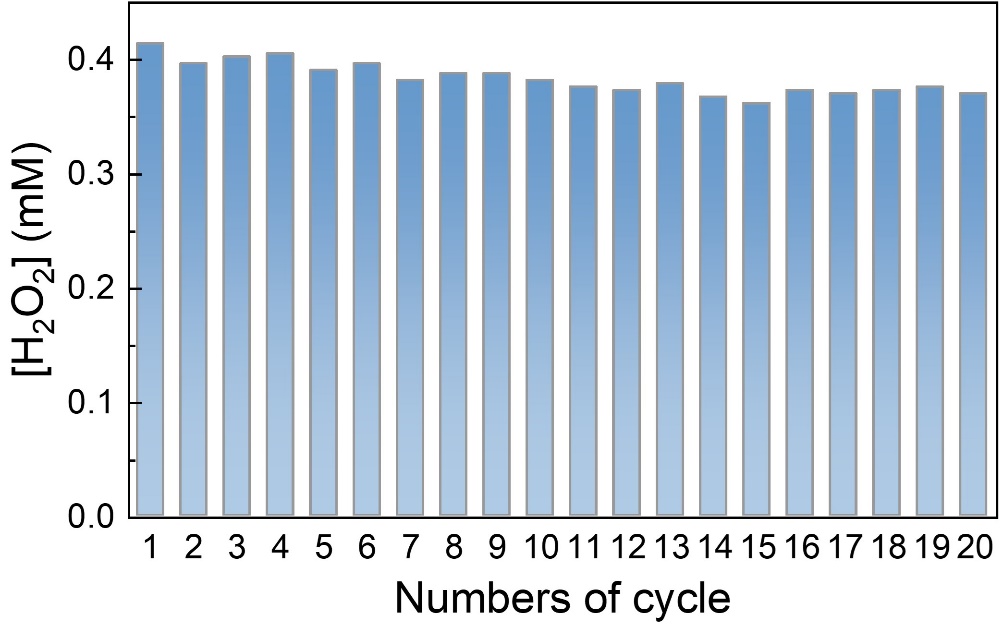


**Fig. S9** The activity performance of SSPM in 20-cyclic experiment


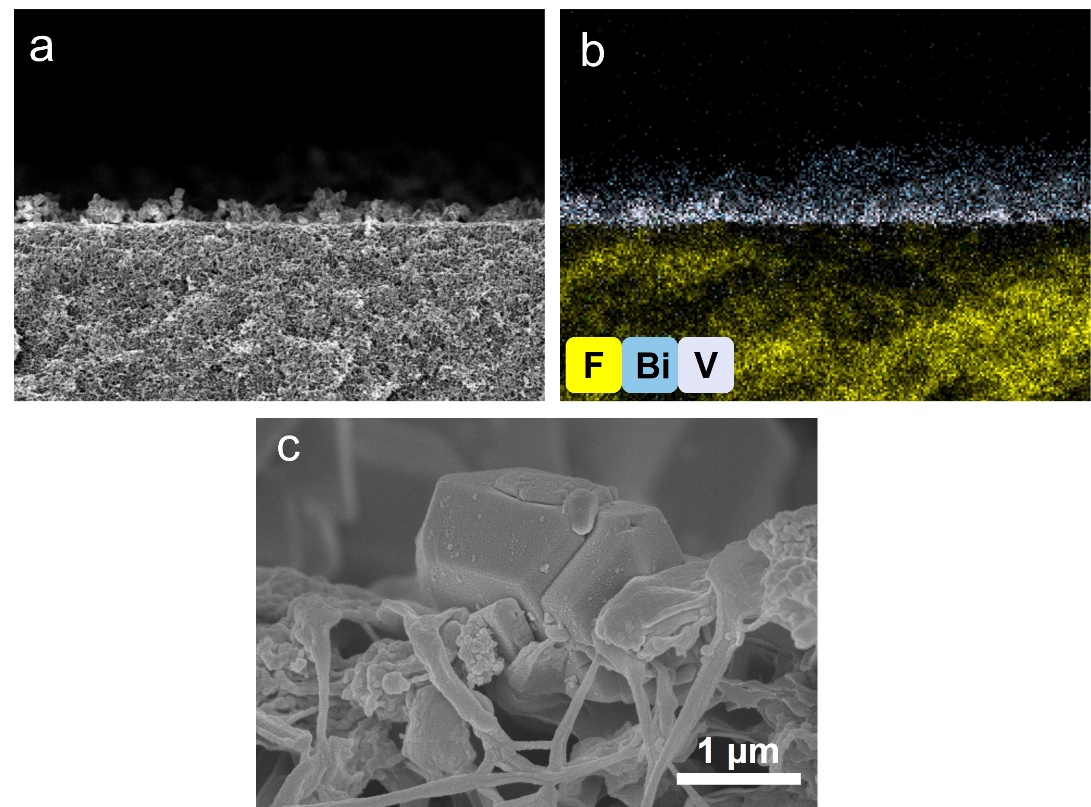


**Fig. S10** SEM image (**a**) EDS mapping (**b**) and high-resolution SEM image of the SSPM after cyclic experiment


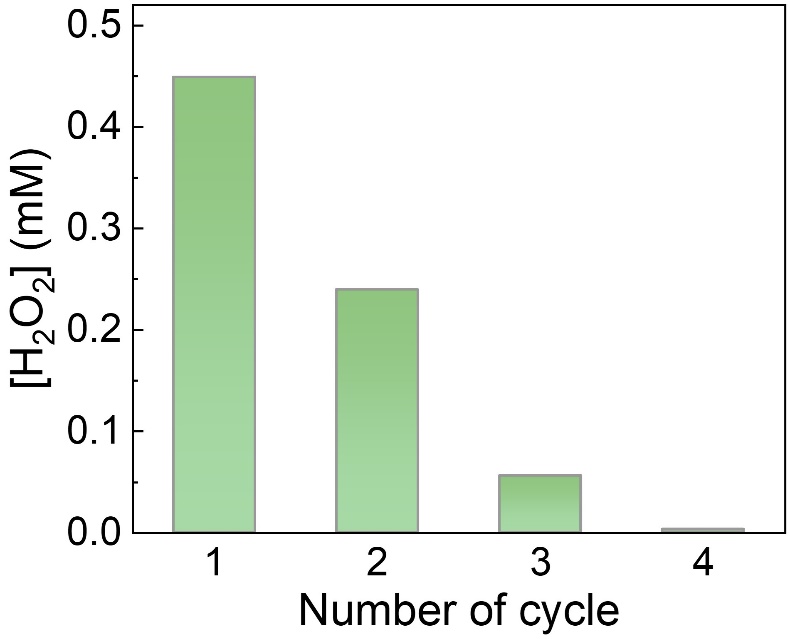


**Fig. S11** The cyclic activity of powder photocatalytic. The initial experiment contains: 10 mg CoO_x_/Mo:BiVO_4_/Pd, 30 mL DI water saturated with O_2_; Xenon lamp solar simulator with AM 1.5G filter, 100 mW/cm^2^


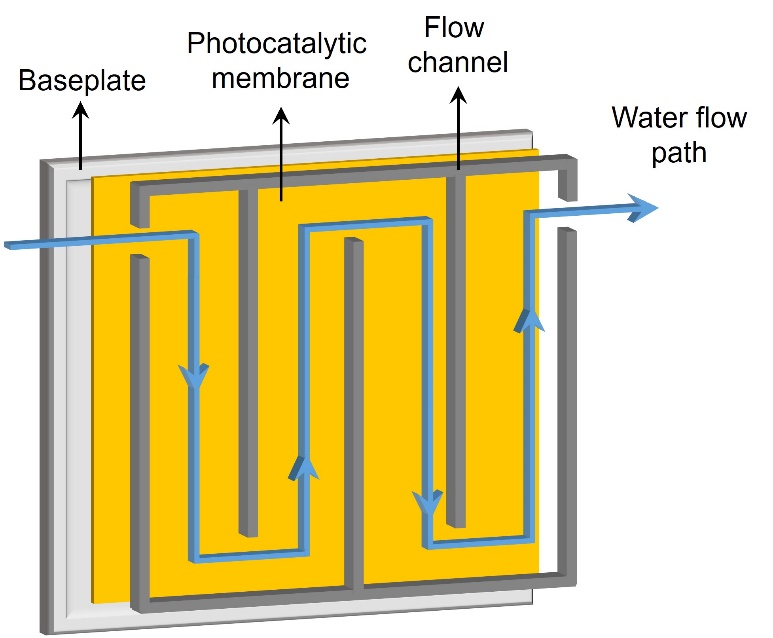


**Fig. S12** Illustration of the structure and water flow pattern of the membrane cell, which consisted of baseplate, photocatalytic membrane and flow channel. The membrane was placed on the baseplate and water flew by the membrane surface and through the serpentine channel


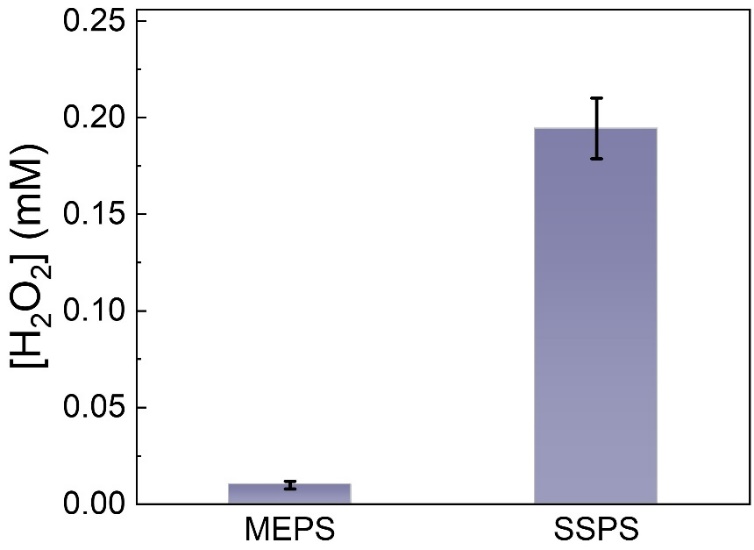


**Fig. S13** H_2_O_2_ generated by MEPM and SSPM in flow reactor at water flow velocity of 0.2 mL/min


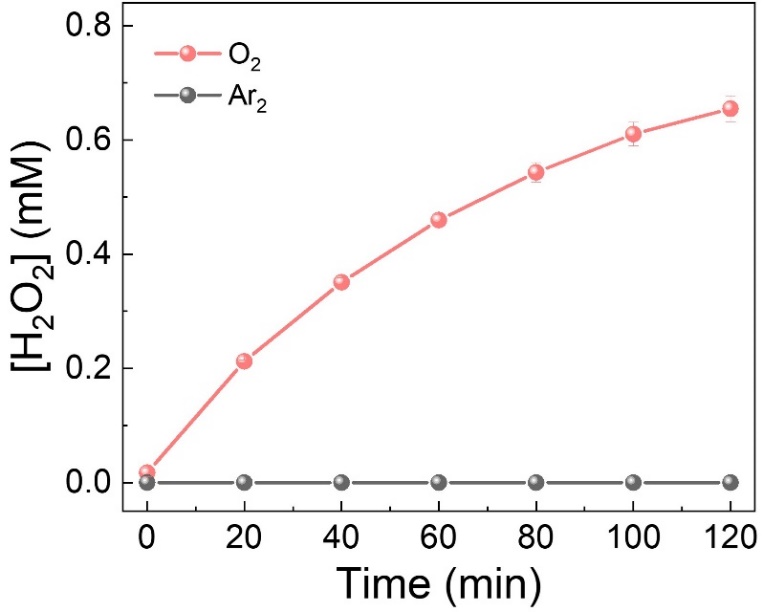


**Fig. S14** H_2_O_2_ photosynthesis performance of SSPM under O_2_ and Ar_2_ purging


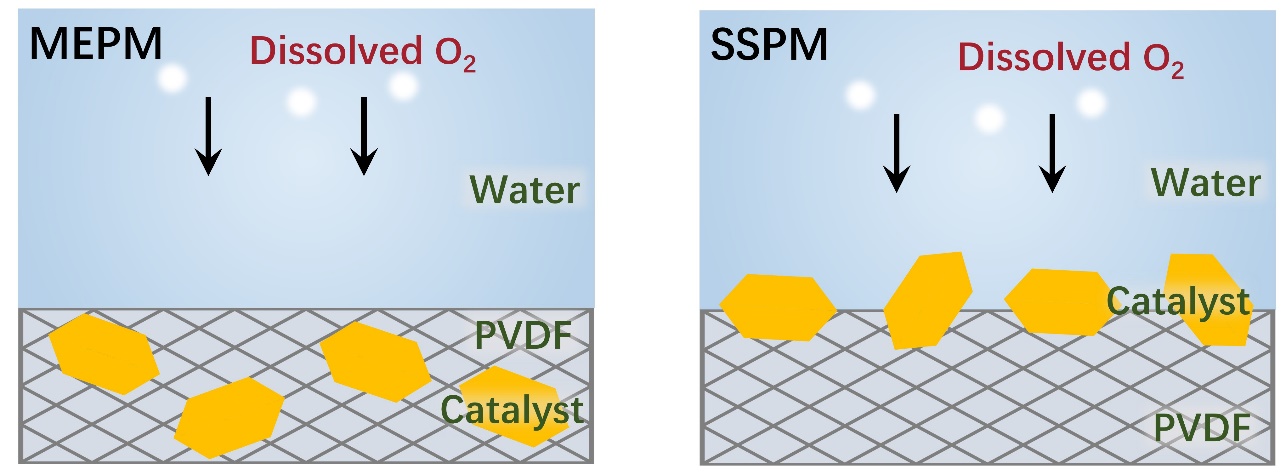


**Fig. S15** Simulation model of O_2_ distribution comprised a free water phase and a porous PVDF substrate. The catalyst was positioned within the substrate for the MEPM and on its surface for the SSPM as shown in the schematic


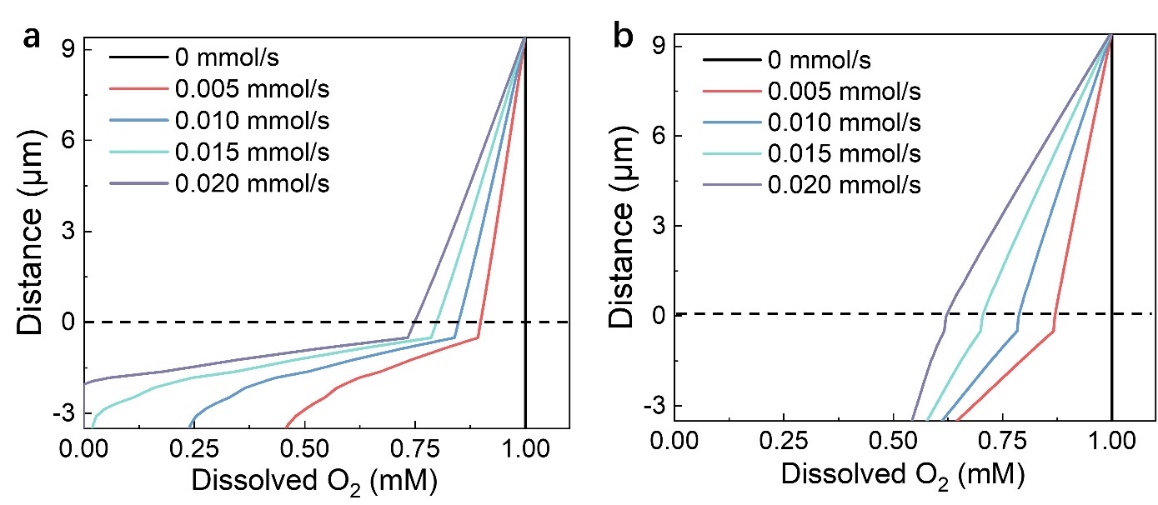


**Fig. S16** O_2_ concentration profile with the Y-axis under different O_2_ consumption rate in (**a**) MEPM and (**b**) SSPM simulated models


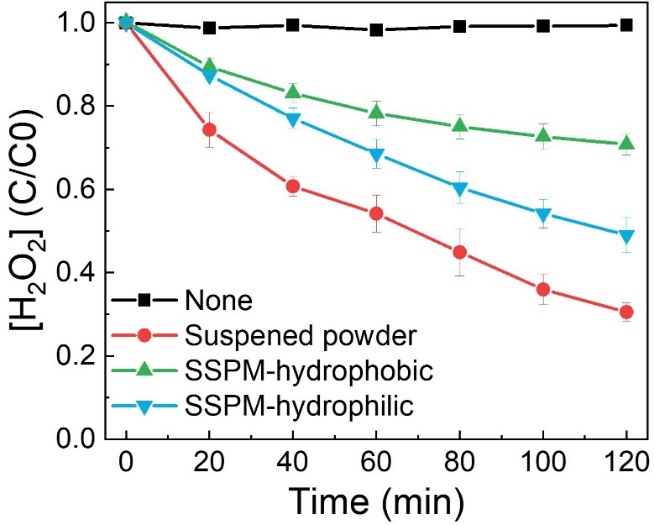


**Fig. S17** The rates of H_2_O_2_ decomposition over blank control, powder suspension, surface bonded sheet with hydrophobic substrate, surface bonded sheet with hydrophilic substrate. The experiment group contains: 2 mg CoO*x*/Mo:BiVO_4_/Pd, 30 mL, 1 Mm H_2_O_2_ solution saturated with N_2_; Xenon lamp solar simulator, 100 mW/cm^2^, AM 1.5G


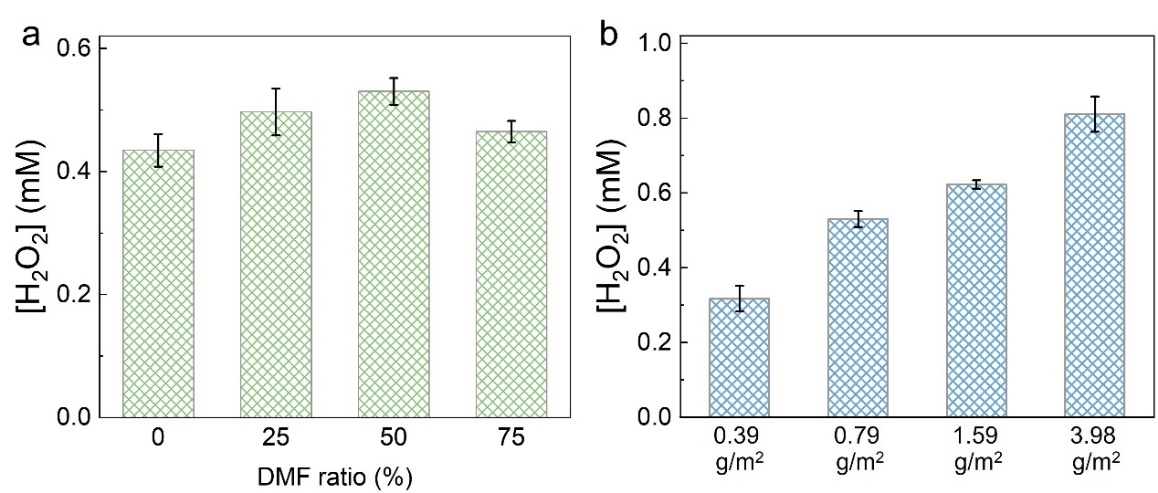


**Fig. S18** The photocatalytic performance of SSPM with different DMF/ethanol ratio (**a**) and catalyst loading amounts (**b**). The optimized DMF/ethanol ratio and catalyst loading amount were 50 % and 3.98 g/m^2^, respectively


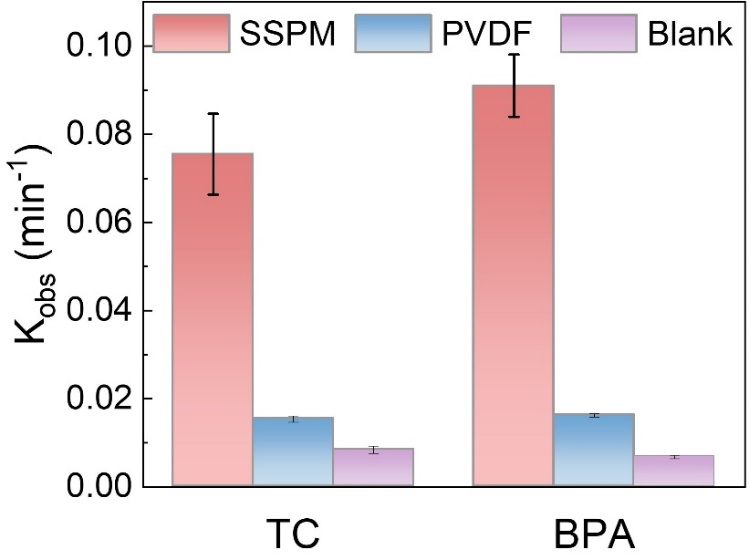


**Fig. S19** The pollutant degradation rates of different photocatalytic membranes under UV light


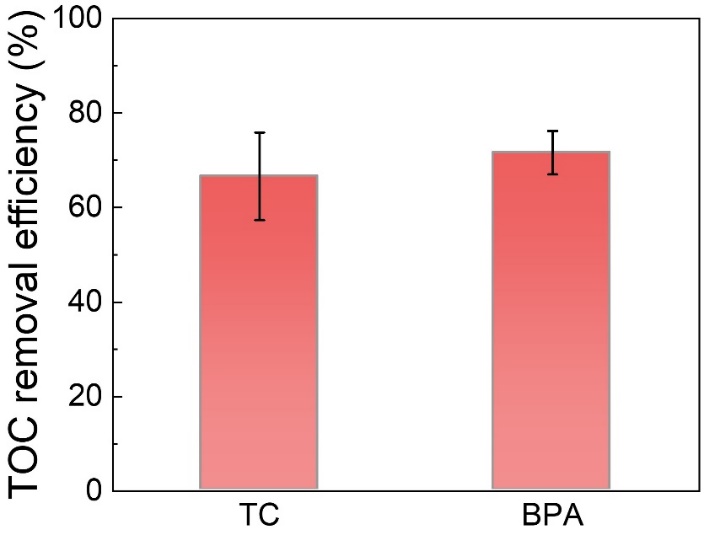


**Fig. S20** TOC removal efficiency of TC and BPA by UV/SSPM treatment in 3 hours
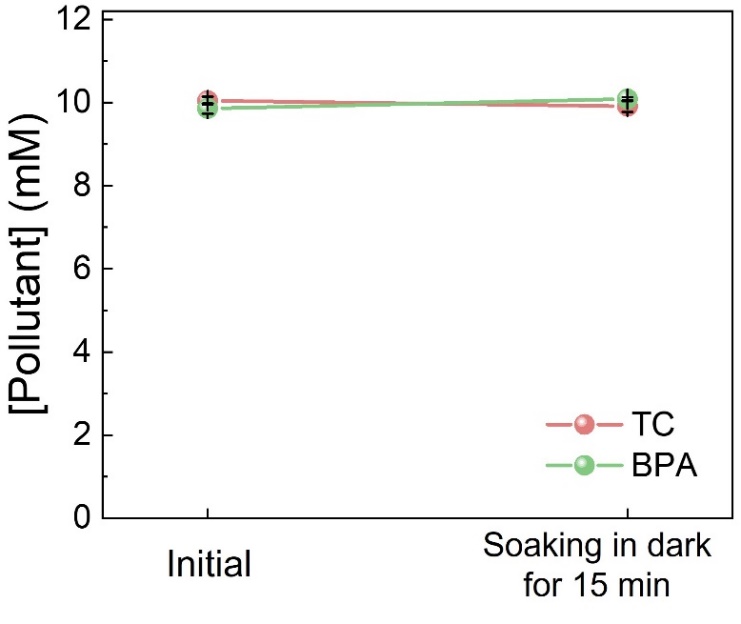


**Fig. S21** The pollutants (TC and BPA) concentration changes when SSPM soaking under dark conditions


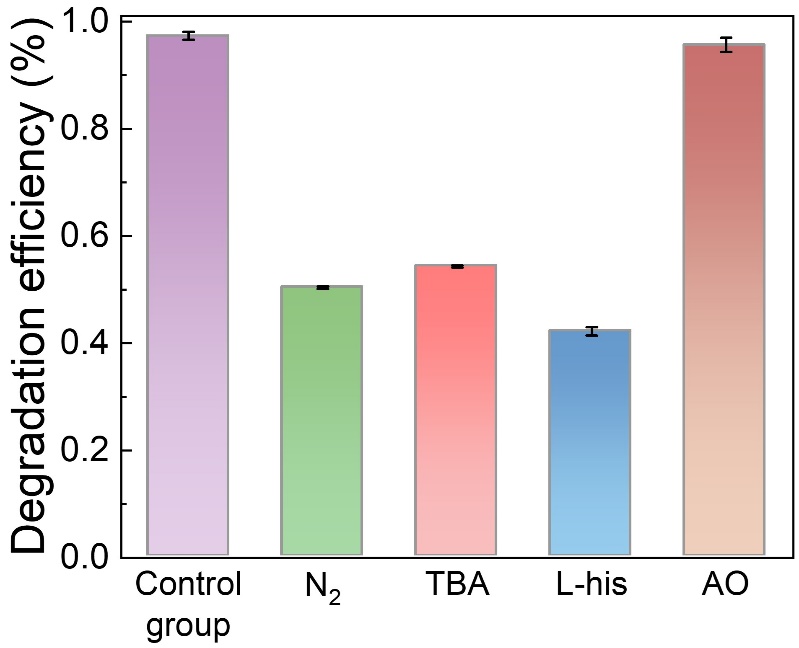


**Fig. S22** The effect of scavengers (N_2_, TBA, L-his and AO) in pollutant degradation experiments ([BPA] = 10 mg/L)


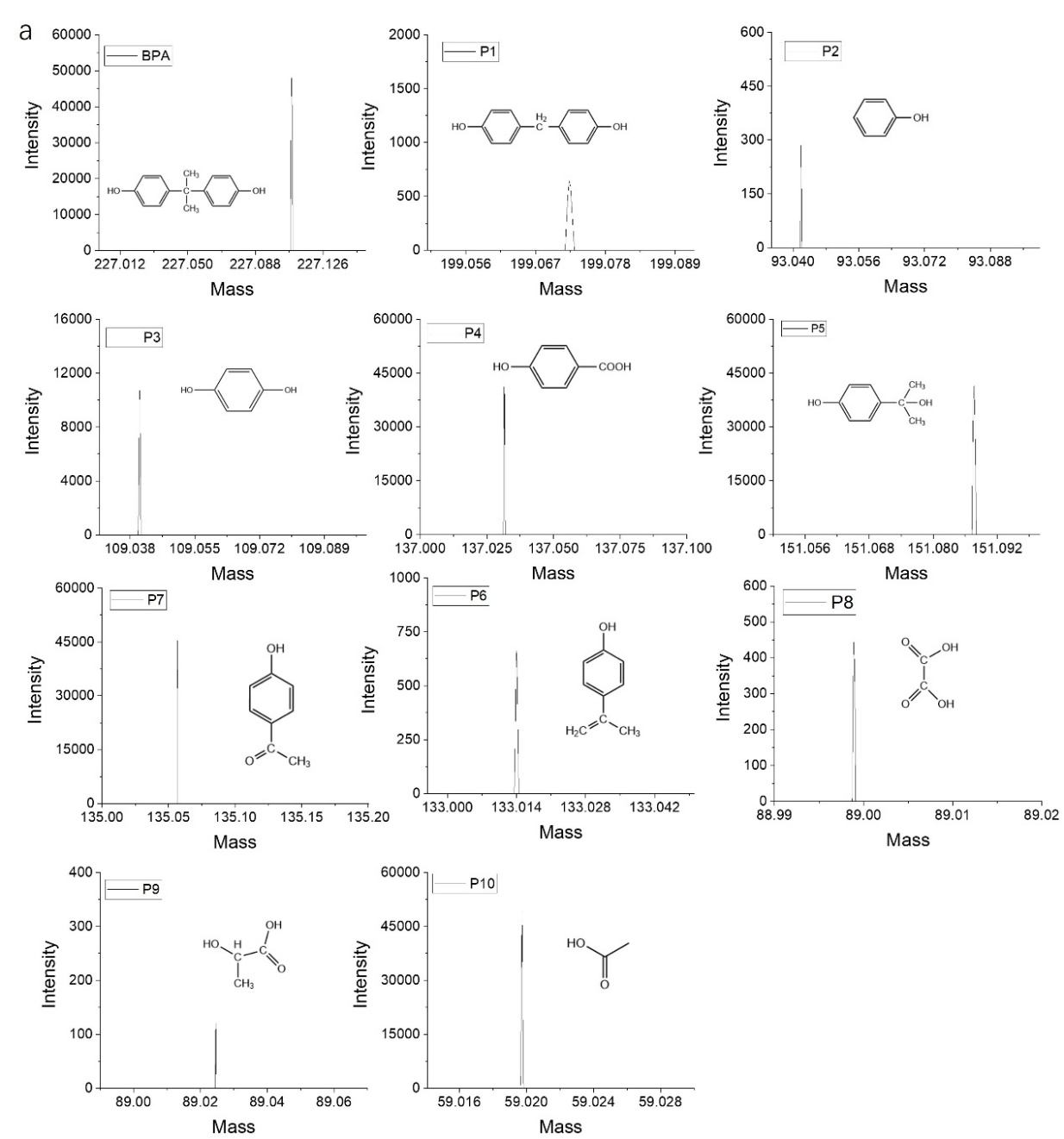


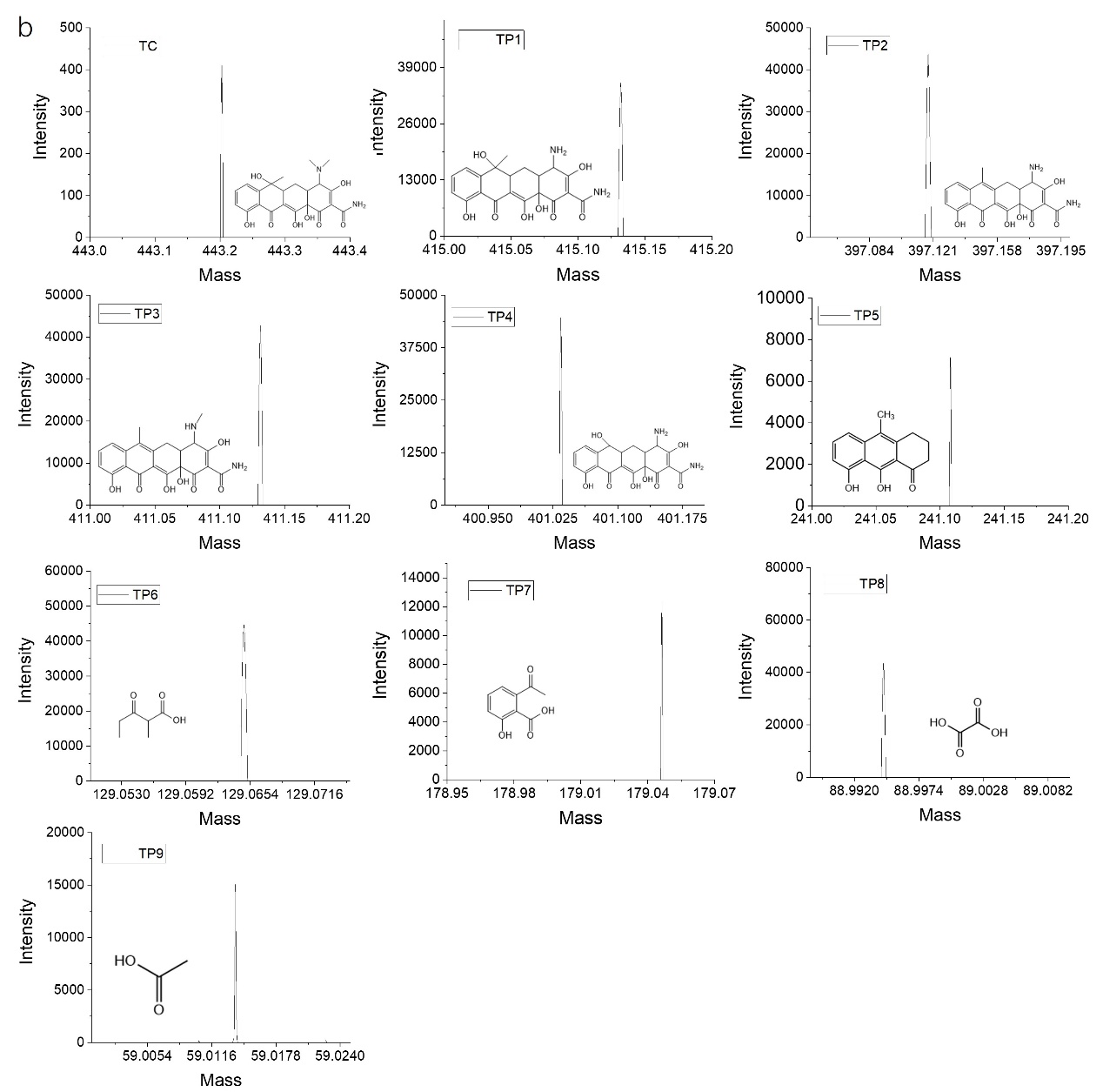


**Fig. S23** The mass spectra of (**a**) BPA and its intermediates; (**b**) TC and its intermediate


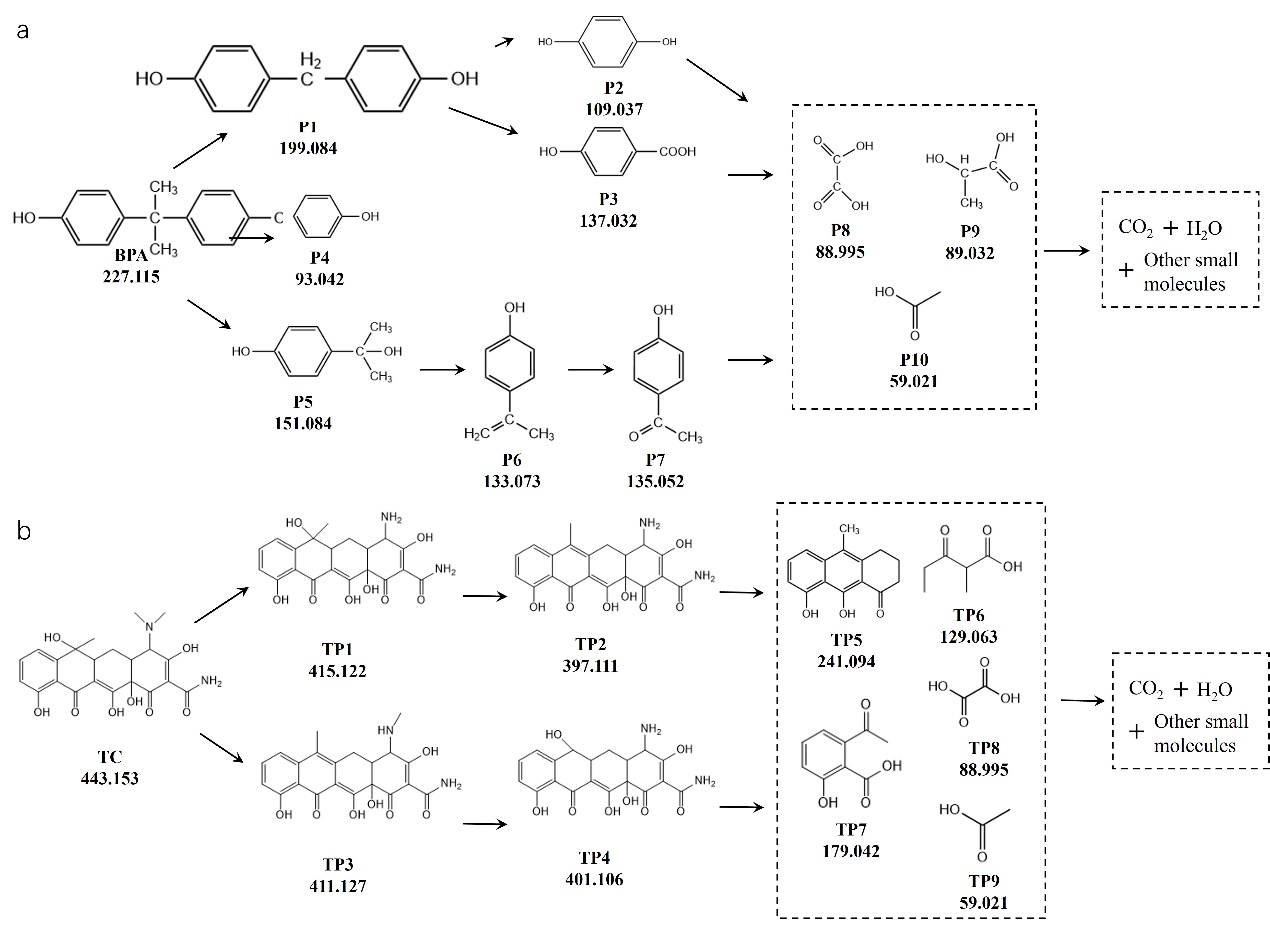


**Fig. S24** The proposed mechanism for the transformation of (**a**) BPA and (**b**) TC based on the degradation intermediates detected


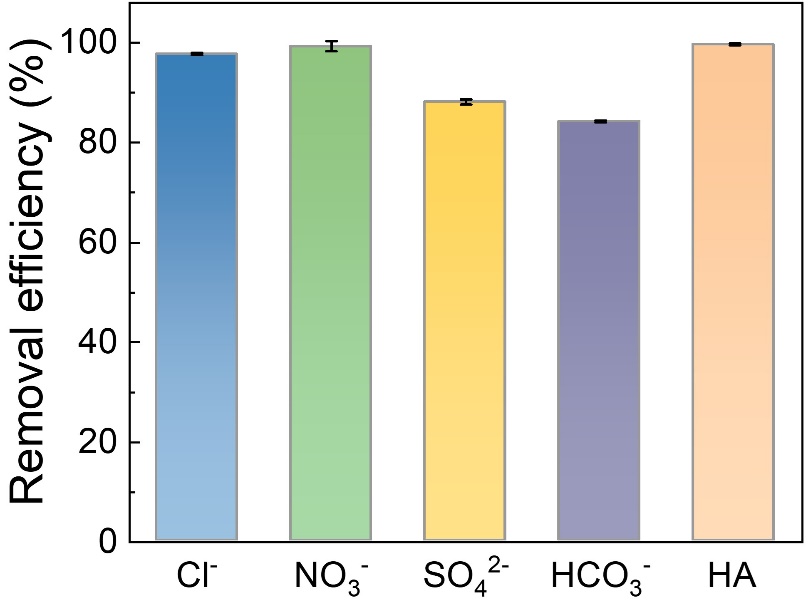


**Fig. S25** The influence of interference components (Cl^-^, NO_3_^-^, SO_4_^2-^, HCO_3_^-^ and humic acid) on pollutant degradation


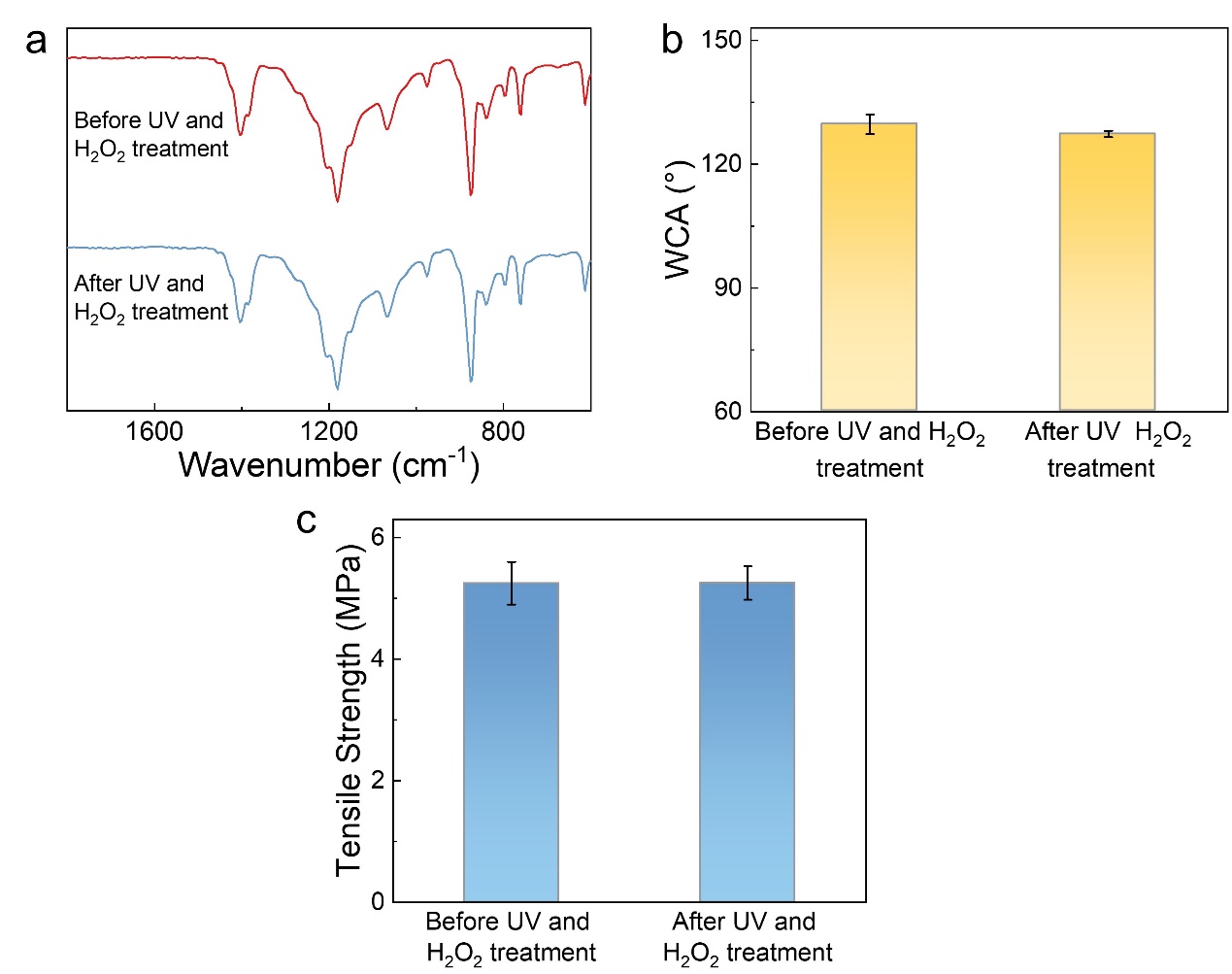


**Fig. S26** (**a**) FTIR spectra of SSPM before and after UV-H_2_O_2_ treatment. (**b**) The water contact angle (WCA) of SSPM before and after UV-H_2_O_2_ treatment. (**c**) The tensile strength of SSPM before and after UV-H_2_O_2_ treatment


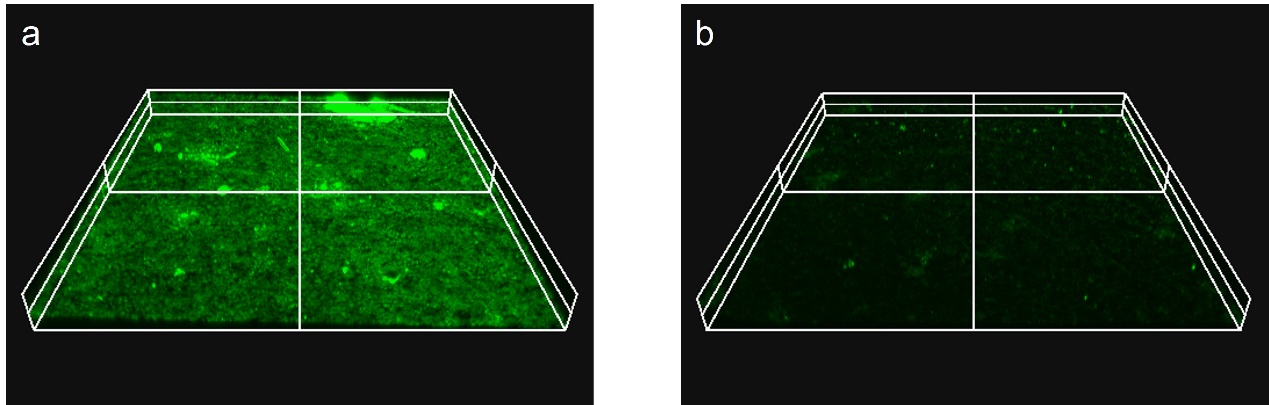


**Fig. S27** The visual membrane surface fouling of PVDF and SSPM membrane. The membrane worked in the lake water for 24 hours and was characterized by Confocal laser scanning microscopy (CLSM)


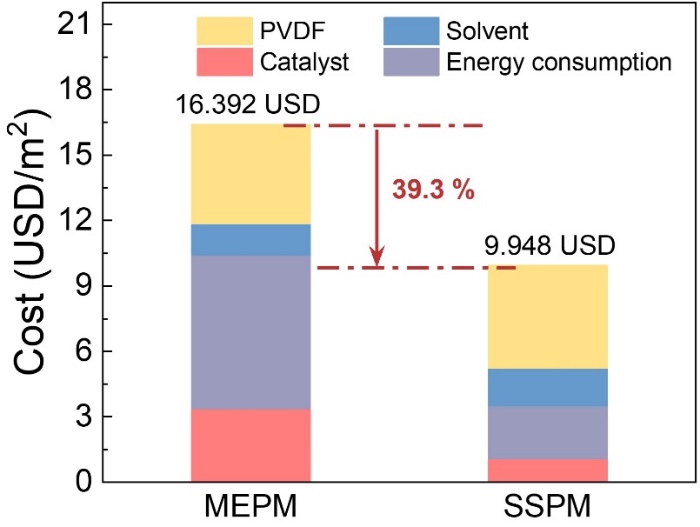


**Fig. S28** The estimated costs for fabricating preparing MEPM and SSPM with each component cost of the photocatalytic sheets, which the SSPM cost (9.948 USD/m^2^ ) was 39.3 % lower than MEPS (16.392 USD/m^2^). The solvent contained the used DMF and ethanol for preparing catalytic sheet


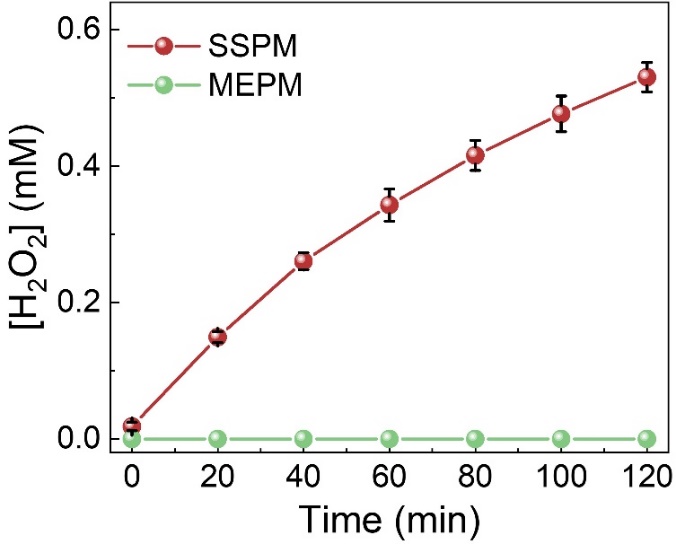


**Fig. S29** The photocatalytic performance of SSPM and MEPM in dry state


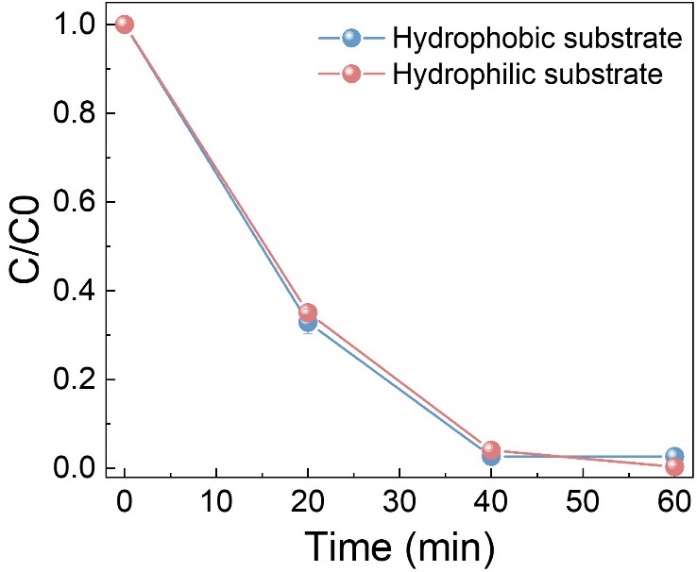


**Fig. S30** The pollutant removal (BPA) performance of SSPM with hydrophobic and hydrophilic substrates. The initial pollutant concentration was 10 mg/L


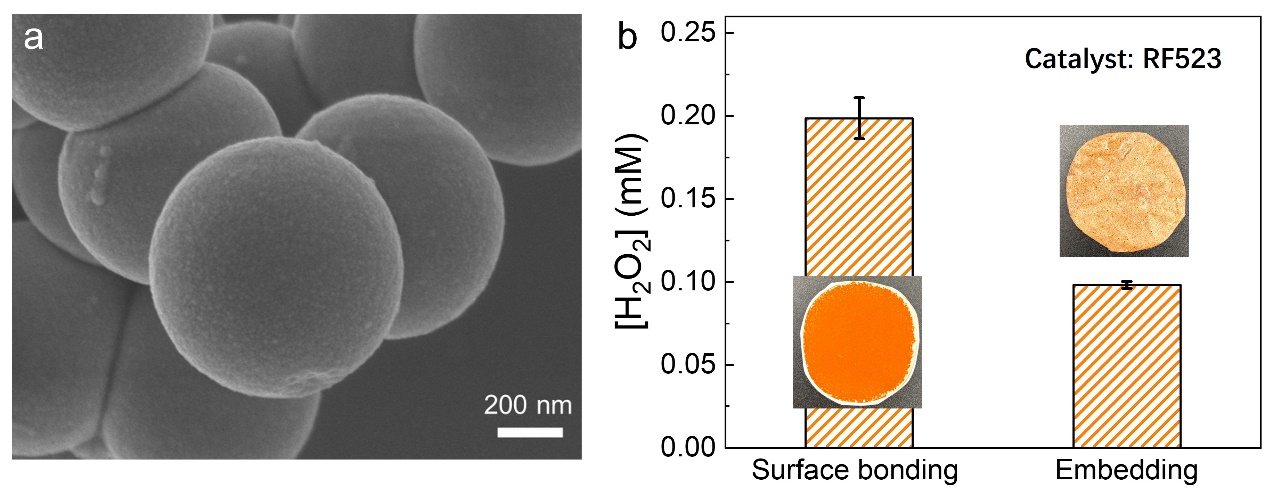


**Fig. S31** (**a**) The SEM of RF 523 catalyst particulate. (**b**) The photocatalytic performance of RF523 in surface bonded and embedded sheets. The insert photos are surface bonded and embedded with RF523 respectively. Experiment condition: 2 mg RF523, 30 mL DI water saturated with O_2_; Xenon lamp solar simulator, 100 mW/cm^2^, AM 1.5G, reaction time is 2h

**Supplementary References**

1. L. Liu, M.-Y. Gao, H. Yang, X. Wang, X. Li et al., Linear conjugated polymers for solar-driven hydrogen peroxide production: the importance of catalyst stability. J. Am. Chem. Soc. **143**(46), 19287–19293 (2021). <https://doi.org/10.1021/jacs.1c09979>
2. X. Zhang, H. Su, P. Cui, Y. Cao, Z. Teng et al., Developing Ni single-atom sites in carbon nitride for efficient photocatalytic H_2_O_2_ production. Nat. Commun. **14**(1), 7115 (2023). <https://doi.org/10.1038/s41467-023-42887-y>
3. J. Hou, K. Wang, X. Zhang, Y. Wang, H. Su et al., Synergistic defect sites and CoO*_x_* nanoclusters in polymeric carbon nitride for enhanced photocatalytic H_2_O_2_ production. ACS Catal. **14**(14), 10893–10903 (2024). <https://doi.org/10.1021/acscatal.4c00334>
4. C. Feng, J. Luo, C. Chen, S. Zuo, Y. Ren et al., Cooperative tungsten centers in polymeric carbon nitride for efficient overall photosynthesis of hydrogen peroxide. Energy Environ. Sci. **17**(4), 1520–1530 (2024). <https://doi.org/10.1039/D3EE03032F>
5. Y. Isaka, Y. Kawase, Y. Kuwahara, K. Mori, H. Yamashita, Two-phase system utilizing hydrophobic metal–organic frameworks (MOFs) for photocatalytic synthesis of hydrogen peroxide. Angew. Chem. Int. Ed. **58**(16), 5402–5406 (2019). <https://doi.org/10.1002/anie.201901961>
6. Y. Zhang, C. Pan, G. Bian, J. Xu, Y. Dong et al., H_2_O_2_ generation from O_2_ and H_2_O on a near-infrared absorbing porphyrin supramolecular photocatalyst. Nat. Energy **8**(4), 361–371 (2023). <https://doi.org/10.1038/s41560-023-01218-7>
7. Q. Wu, J. Cao, X. Wang, Y. Liu, Y. Zhao et al., A metal-free photocatalyst for highly efficient hydrogen peroxide photoproduction in real seawater. Nat. Commun. **12**(1), 483 (2021). <https://doi.org/10.1038/s41467-020-20823-8>
8. L. Li, L. Xu, Z. Hu, J.C. Yu, Enhanced mass transfer of oxygen through a gas–liquid–solid interface for photocatalytic hydrogen peroxide production. Adv. Funct. Mater. **31**(52), 2106120 (2021). <https://doi.org/10.1002/adfm.202106120>
9. R. Liu, Y. Chen, H. Yu, M. Položij, Y. Guo et al., Linkage-engineered donor–acceptor covalent organic frameworks for optimal photosynthesis of hydrogen peroxide from water and air. Nat. Catal. **7**(2), 195–206 (2024). <https://doi.org/10.1038/s41929-023-01102-3>
